# Control the System and Environment of Post-Production Wind Turbine Blade Waste Using Life Cycle Models. Part 1. Environmental Transformation Models

**DOI:** 10.3390/polym12081828

**Published:** 2020-08-14

**Authors:** Izabela Piasecka, Patrycja Bałdowska-Witos, Józef Flizikowski, Katarzyna Piotrowska, Andrzej Tomporowski

**Affiliations:** 1 Faculty of Mechanical Engineering, University of Science and Technology in Bydgoszcz, 85-796 Bydgoszcz, Poland; fliz@utp.edu.pl (J.F.); a.tomporowski@utp.edu.pl (A.T.); 2 Faculty of Mechanical Engineering, Lublin University of Technology, 20-618 Lublin, Poland; k.piotrowska@pollub.pl

**Keywords:** CED, Eco-indicator 99, IPCC, LCA, waste, wind energy, wind power plant blade, recycling

## Abstract

Controlling the system—the environment of power plants is called such a transformation—their material, energy and information inputs in time, which will ensure that the purpose of the operation of this system or the state of the environment, is achieved. The transformations of systems and environmental inputs and their goals describe the different models, e.g., LCA model groups and methods. When converting wind kinetic energy into electricity, wind power plants emit literally no harmful substances into the environment. However, the production and postuse management stages of their components require large amounts of energy and materials. The biggest controlling problem during postuse management is wind power plant blades, followed by waste generated during their production. Therefore, this publication is aimed at carrying out an ecological, technical and energetical transformation analysis of selected postproduction waste of wind power plant blades based on the LCA models and methods. The research object of control was eight different types of postproduction waste (fiberglass mat, roving fabric, resin discs, distribution hoses, spiral hoses with resin, vacuum bag film, infusion materials residues, surplus mater), mainly made of polymer materials, making it difficult for postuse management and dangerous for the environment. Three groups of models and methods were used: Eco-indicator 99, IPCC and CED. The impact of analysis objects on human health, ecosystem quality and resources was controlled and assessed. Of all the tested waste, the life cycle of resin discs made of epoxy resin was characterized by the highest level of harmful technology impact on the environment and the highest energy consumption. Postuse control and management in the form of recycling would reduce the negative impact on the environment of the tested waste (in the perspective of their entire life cycle). Based on the results obtained, guidelines and models for the proecological postuse control of postproduction polymer waste of wind power plants blades were proposed.

## 1. Introduction

Human economic activity consists of using the natural resources of the surrounding nature, which at the same time is a place of introducing pollution and waste resulting from processing natural resources into useful materials, products, energy carriers, machinery, equipment, construction and industrial facilities, etc. Some problems with the scope of environmental protection do not constitute a direct threat to human health, but they have a significant impact on reducing the quality of life (e.g., noise, dust, excessive traffic intensity, etc.). In industrial activities related to the production of energy, products and services, many different environmental burdens arise, which include both the use of natural resources and the introduction of pollution causing slow degradation of all elements of the environment [1,2,3].

Conventional energy uses huge amounts of fossil fuels (e.g., coal, oil, gas), and their processing processes cause this sector of the economy to have the largest share in emissions of environmental pollutants, in particular into the atmosphere. The introduction of pollutants into the environment takes place mainly in the processes of obtaining conventional fuels and their combustion [4,5].

**Figure 1:**
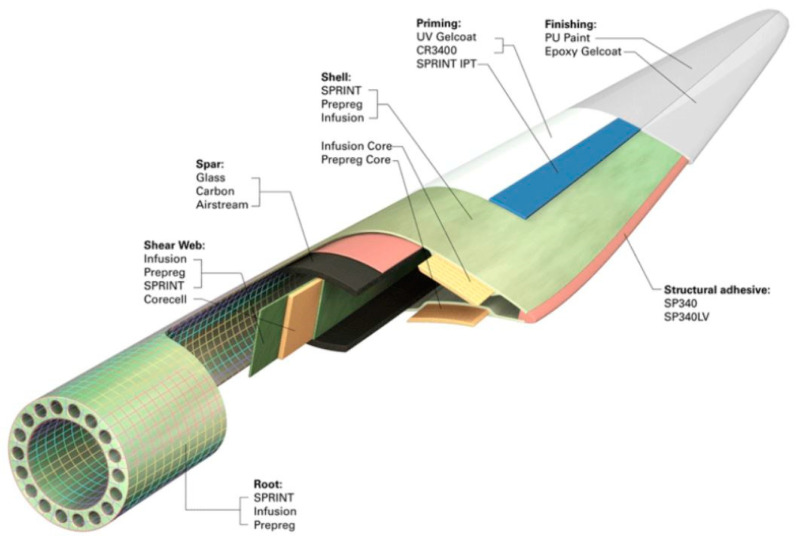
Cross-section of wind power plant blade, including the most important materials and components [10].

Wind power plants are considered as a “clean” energy source that meets the principles of sustainable development. Although the production of electricity from wind energy is associated, for example, with lower CO_2_ emissions than in the case of fossil fuel power plants, their waste cycle also generates a lot of waste and emissions (mainly at the stage of production and postuse management). The majority of high-power wind power plants is steel, mainly used for tower production. Steel is usually recycled, which does not cause major difficulties, due to the well-known technology of this process. However, wind power plant blades are made of lightweight, composite polymer materials, which are characterized by a large volume and a number of difficulties during recycling. Although the weight of the blades is limited compared to the total weight of the wind power plant, it is significant (one blade usually weighs over 6–7 tons), especially taking into account the effect of the scale of wind energy development. The construction of an example wind power plant blade is shown in Figure 1 [6,7,8,9].

The European Waste Framework Directive (2008/98/EC) defines the most important concepts related to waste management. The wind energy industry is increasingly implementing the ideas of sustainable management of plastics, materials and postconsumer elements. According to the hierarchy in the directive, the most important thing is waste prevention. In this regard, activities at the stage of designing the blades of wind power plants are of key importance, including taking into account the reduction in their weight (less material for postuse disposal), reduction in failure rate, extended design lifetime or innovative structures (for example: segmented/modular blades). However, these are not standard designs. According to the directive, blades of wind power plants should be used for as long as possible, hence the importance of the issue of their reuse. Each day there are more companies dealing with refurbished turbines and components on the market. Repurposing holds another place in the waste management hierarchy. It means reusing the blades of wind turbines for other purposes, for example, as elements of playgrounds, bicycle shelters or elements of small architectural constructions. However, this is not a comprehensive solution taking into account the number of blades of wind power plants decommissioned (these are rather demonstration projects). If repurposing is not possible, recycling and recovery applies. Disposing blades via landfill or incineration without energy recovery is the least preferred method of postutility management, because it does not provide material nor energy recovery.

Today, the most commonly used method of recycling composite waste is through cement coprocessing (“the cement kiln route”). Composites can also be developed as postconsumer elements through mechanical grinding, thermal (pyrolysis, fluidized bed), thermo-chemical (solvolysis), or electro-mechanical (high voltage pulse fragmentation) processes or combinations of these. However, not all of these methods are currently adapted for use on an industrial scale. Different processing methods have a different effect on the fiber quality (length, strength, stiffness properties), which translates into the subsequent possibility of economic use of the recycled fibers. Unfortunately, in most cases, recycled material is much more expensive than virgin materials. Nowadays, the best strategy for the postuse development of wind power plant blades is combined design, maintenance, upgrades (for example reinforcement) and the appropriate recycling technology. It should additionally take into account the reuse of materials for the same or similar purposes (for example, allows polymer matrices to revert to monomers and avoids fiber damage during the process). A more detailed description of the methods of the postuse management of wind power plant blades can be found, among others, in works [11,12,13,14]. Understanding the environmental impacts associated with the appropriate selection of materials associated with different postuse management methods when designing, in the context of the lifecycle of wind power plant blades, will play a key role in determining a sustainable development strategy in this area.

Interest in environmental protection issues, in particular, the combination of ecological issues with economic decisions, requires consideration of the potential impact on the environment of products and services. In the entire sphere of economic activity, the aim is to minimize the impact on the environment, perceived not only in relation to a single aspect of impact but impacts in a comprehensive manner. Dynamic changes in economic conditions and social expectations mean that in the life cycle of business operations, including those producing wind power plants blades, in addition to production and construction aspects, environmental protection objectives should be taken into account. To this end, appropriate assessment tools are needed to take account emerging environmental problems. The basic tool used for such assessment is the energy and ecological analysis of products, covering their entire life cycle—known as the LCA (Life Cycle Assessment) methodology. It can be successfully used to evaluate production, operating or postuse management processes, included by adopting the level of depletion of natural resources and the impact of pollution as assessment criteria [15,16,17,18].

Thanks to the introduction of ISO 14040 (Environmental management, Life cycle assessment, Principles and framework) and ISO 14044 (Environmental management, Life cycle assessment, Requirements and guidelines) standards, the LCA methodology is nowadays an assessment tool, ensuring comparable analysis results, while considering many important ecological problems. It introduces order when considering complex issues related to the assessment of the impact on the environment, taking into account the entire life cycle of research objects. It has gained international recognition and is widely regarded as an effective tool for assessing the environmental impact of objects [3,18].

All products and services exert some influence on the environment, which may occur at one, several or all stages of their life cycle—from raw material sourcing, production, distribution and operation, to various forms of postuse management. These interactions can be very diverse, may have different intensities and last for different periods of time; they can also have different ranges [18,19].

Energetic system and ecological control analyses were carried out in order to answer the question about potential damage to the environment as a result of the lifecycle of a given object, for example wind, power plant blades. Impacts resulting from both the acquisition and use of resources as well as the generation of pollution and waste and their introduction into the environment have an important meaning. If the analysis does not fully answer the question posed in this way, it will at least allow the assessment of what potential environmental threats resulting from the lifecycle of the object can be expected. For this purpose, it is necessary to link the processes occurring at every stage of the life cycle with the damage carried to the environment, in particular, in areas of environmental protection, which include human and animal health, environmental quality, protection of agricultural crops and forests, natural resources or products anthropogenic [17,20,21,22].

Therefore, the main goal of this analysis was ecological and energetic environment and system control analysis of the life cycle of selected postproduction waste of wind power plant blades.

Such an extremely multifaceted topic as ecological and energetic environment and system control analysis of the life cycle of selected postproduction waste of wind power plant blades could not be fully discussed in this article, regardless of its large volume. It seems reasonable to develop the discussed issues in the form of the second part of the article, covering the most important environmental aspects in the field of grouping and weighing (nonobligatory phases) in the LCA method.

## 2. Models and Methods

### 2.1. Object and Plan of Analysis

The object of environment and system control analysis was eight types of postproduction waste generated during the production of wind power plant blades, 49 m long, designed for 2 MW wind power plant. Wastes accounted for were: fiberglass mat, roving fabric, resin discs, distribution hoses, spiral hoses with resin, vacuum bag film, infusion materials residues and surplus materials. They were selected for research because they have the largest mass participation in all waste that arises during the production of wind power plant blades and cause difficulties during subsequent postuse management, due to their structure and materials from which they were made (mainly polymeric materials).

**Figure 2:**
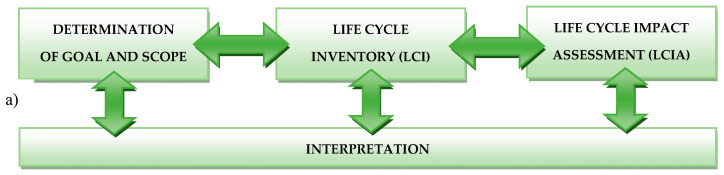

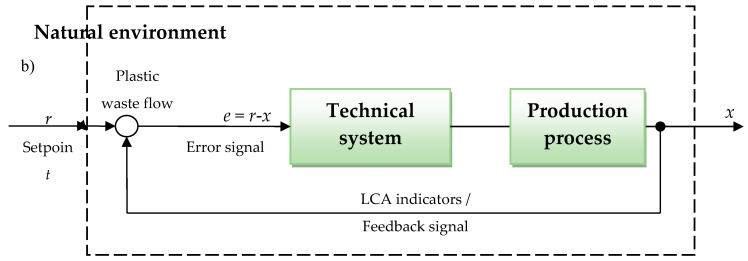
Stages of LCA analysis (in accordance with ISO 14044 (**a**) and flowchart of the closed arrangement of the environment and wind power plants energy system (**b**).

LCA—Life Cycle Assessment was chosen as the main method of assessing the potential impact on the postproduction waste environment of wind power plant blades. According to ISO 14044 standards, LCA analysis should include four stages: determination of goal and scope, life cycle inventory (LCI), life cycle impact assessment (LCIA) and interpretation (Figure 2) [23,24].

A system with one input *u* (components) and one output *x* (products) was considered [25]. The functions *u* (*t*) and *x* (*t*) are defined for each *t*. It was assumed that the input u of the system (blade production) from Figure 2 is also the output of another system (environmental resources and system waste). If the latter system is a combination of the controller and an actuator, such as, for example, a control model, its input may be the error signal *e*. This signal is the difference between the setpoint *r* and the output value *x* of the system. Environmental and technical system control was considered as a continuous composition reactor. If the flow rate of plastics waste is disturbed, it affects the course of the reaction and ultimately load on the environmental or technical system.

In accordance with ISO 14040 and 14044 standards, the analysis carried out as part of this study included four stages. During the first stage, the purpose and scope of the analysis was formulated, and its details are presented in Section 2.2. Its source came from the analysis of the current state of knowledge and technology, which showed the lack of detailed energy and environmental studies of waste generated during the production of wind power plant blades. The basis for formulating the purpose and scope was also the collection of the largest possible amount of the highest quality data on research objects. It was possible thanks to cooperation with one of the largest wind power plant blades companies in the world. Detailed information on the second stage of analysis, i.e., the LCI, is provided in Section 2.3. The third stage consisted of conducting a detailed life cycle analysis of the eight considered types of postproduction waste of the wind power plant blades. SimaPro 8.4 software was used for the simulations. The calculation procedures were the Eco-indicator 99, IPCC (Intergovernmental Panel on Climate Change, Global Warming Potential) and CED (Cumulative Energy Demand) methods. The course of the third stage is approximated in Section 2.4. All of the above-mentioned results (together with a discussion) are listed in Section 3.1 (Eco-indicator 99), Section 3.2 (IPCC) and Section 3.3 (CED), in turn. The last stage, characterized in Section 2.5, consisted of the interpretation of the results obtained. Its details are in both Section 3.1, Section 3.2, Section 3.3 and Section 4 [26,27,28].

### 2.2. Determination of Goal and Scope

In LCA, the scope of analysis includes: functional unit, system boundaries, assumptions and limitations, data quality requirements and impact categories. This paper evaluated eight elements (eight types of postproduction waste) generated during the production of wind power plant blades. It was possible to collect high-quality data on tested objects thanks to cooperation with one of the largest wind power plant blade companies in the world. Samples of postproduction waste as well as data on blade manufacturing processes were obtained. The conducted research was aimed at a detailed, ecological and energetic control analysis of the life cycle of selected postproduction waste. The completed procedure was a classic LCA process, in accordance with the guidelines contained in ISO 14044 standards, and its main task was to determine the level of negative impact of the life cycle of the analyzed objects on human and animal health, environmental quality and depletion of natural resources. Life cycles and the magnitude of the impact of recycling processes were assessed in detail in fiberglass mat, roving fabric, resin discs, distribution hoses, spiral hoses with resin, vacuum bag film, infusion materials residues and surplus materials. The environmental assessment included eleven impact categories available under the Eco-indicator 99 model: carcinogens, resp. organics (organic compounds causing respiratory diseases), resp. inorganics (inorganic compounds causing respiratory diseases), climate change, radiation, ozone layer, ecotoxicity, acidification/eutrophication, land use, minerals and fossil fuels. The obtained test results were also compiled for four areas of emissions of individual chemical compounds: air, water, soil and raw. Environmental analyzes were supplemented with research using the IPCC method, which made it possible to assess the impact of the most important greenhouse gases on the total greenhouse effect caused by the cycles of existence of test objects. As part of the conducted energy analyzes, the CED method was used. The obtained results allowed the assessment of energy consumption of processes occurring within the life cycle and during recycling of all types of considered postproduction waste. They were assigned to categories covering nonrenewable (fossil, nuclear) and renewable (biomass, wind, solar, geothermal, water) sources [29,30,31,32].

Most of the processes that take place as part of the lifecycle of tested wind power plant blades take place in Europe. For this reason, the scope of the analysis referred to European conditions and a geographical boundary was recognized as territory of Europe. No specific time horizon was adopted, as waste should be subject to postuse management as soon as possible. The functional unit was defined as the generation of one ton of postproduction waste of a given type (1 mg). The analysis covered almost the entire life cycle of the considered postproduction waste of wind power plant blades (did not include the stages of waste storage and transport to postuse management). The main reason was the different (but usually short) storage period and large differences in the environmental effects of transport processes, including depending on the location of recycling facilities or landfills. The conducted analysis can be classified as a bottom-up type and will serve mainly to describe the existing reality (retrospective analysis), but also to model more pro-environmental solutions (prospective analysis). The level of advancement of the conducted research is classified as a detailed analysis.

### 2.3. Life Cycle Inventory (LCI) of Technical System

To make the data collection easier and precise, special inventory sheets were created. A separate form was assigned to each unit process. Each worksheet included entries and exits to the process data related to its implementation. As part of the entrances, main materials, auxiliary materials and any water demand were included. The outputs included the main product and various types of emissions. In the area of process implementation, however, its duration and the resulting media consumption were taken into account. Most of the data were obtained directly from the wind power plant blades manufacturer. Data related to processes and materials, less important from the point of view of environmental impact, were obtained from the databases contained in the SimaPro 8.4 software (ecoinvent 3.4 database). Due to confidentiality agreements, all detailed information about the facilities analyzed and process data is not disclosed in this publication [26,33,34].

**Figure 3:**
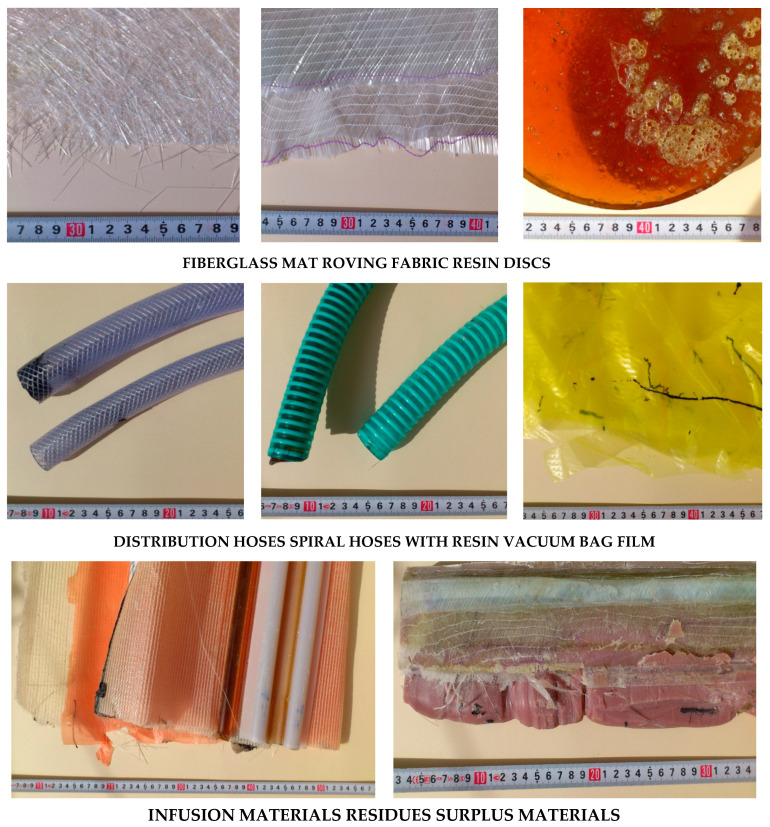
Investigated postproduction waste of wind power plant blades.

Eight types of waste were assessed: fiberglass mat (made of chopped glass fiber), roving fabric (made of epoxy-silane coated fiberglass), resin discs (made of epoxy resin), distribution hoses (inner and outer layer made of high quality PVC material, reinforcement—polyester fiber), spiral hoses with resin (wall made of high plasticized PVC, reinforcement—rigid PVC helix reinforced inside with epoxy resin), vacuum bag film (made of co-extrusion of polyolefin and nylon based resins), infusion materials residues (epoxy resin, infusion mesh made of extruded LDPE grid mesh, release film made of highly flexible polypropylene-based release film, profile channel made of PVC, polyester release fabric made of polyester, flexible mold release tape made of polytetrafluoroethylene and silicone adhesive, sealant tape made of butyl) and surplus materials (polypropylene, epoxy resin, roving fabric made of epoxy silane coated fiberglass, fiberglass mat made of chopped glass fiber, release film made of highly flexible polypropylene-based release film, polyester glue made of polyester fiberglass reinforced resin) (Figure 3) (manufacturer’s data).

After assigning data to unit processes, they were validated by making a two-way energy and mass balance. Models were systematically constructed and filled with subsequent data. The size of the entries balanced the size of the exits. Thanks to this, it was possible to aggregate data as well as convert them into a functional unit and reference streams. By adding together environmental data of the same type (input of materials, energy, emissions, etc.) for all unit processes, input–output matrices were obtained. They were assigned to reference streams, and thus, inventory tables were obtained. Finally, the data were adapted to the format used in SimaPro 8.4. By performing this procedure, it became possible to enter them into the program and move to the third stage of the analysis [26,35].

### 2.4. Life Cycle Impact Assessment (LCIA) of Postproduction Polymer Waste

Life Cycle Impact Assessment consisted of assessing the impact of the lifecycle of postproduction waste of wind power plant blades on the environment. The results of the previous stage (LCI) were associated with indicators for assessing the impact of a given process on the environment, using the impact category and characterization parameter. Impact Assessment included selecting impact category, characterization models, followed by classification, characterization, normalization, grouping and weighting (for details, see Section 2.4.1, Section 2.4.2 and Section 2.4.3). LCIA was made using SimaPro 8.4 software (PRé Sustainability, LE Amersfoort, The Netherlands). The cut-off level was 0.1%. Ecological and energetic analysis of the life cycle of selected postproduction waste of wind power plant blades was performed by using three methods: Eco-indicator 99, IPCC (Intergovernmental Panel on Climate Change) and CED (Cumulative Energy Demand). The results of this stage are approximated in Section 3.

#### 2.4.1. Eco-Indicator 99 Method

The Eco-indicator 99 method belongs to the group of methods modeling the environmental impact at the endpoint level of the environmental mechanism. The characterization process takes place for eleven impact categories, falling into three larger groups known as areas of influence. There are the following areas of influence: human health, ecosystem quality, resources. The results of the areas of influence indicators are further aggregated in the final Ecolabel through normalization, grouping and weighting (Table 1) [30,36,37].

Human health is one of the areas of influence in the Eco-indicator 99 method, which in turn consists of six impact categories: carcinogens, resp. organics, resp. inorganics, climate change, radiation and ozone layer. By defining the area of influence indicator from the endpoints of the environmental mechanism, it is possible to adopt a common unit for all impact categories under human health. Each of them can cause the same type of impact, for example, health disorders in humans and animals. In the Eco-indicator 99 method, as part of the characterization stage, the DALY (Disability Adjusted Life Years) scale developed by Murray for the World Bank and WHO was introduced. Within this framework, various diseases were assigned weights from 0 (ideal health) to 1 (death) [26,38,39].

Within ecosystem quality, three impact categories are distinguished: ecotoxicity, acidification/eutrophication and land use. The area of influence of ecosystem quality is much more diverse and less homogeneous compared to the area of human health. The basis for the occurrence of various phenomena modeled in its scope is often so different to a common unit which has not been clearly defined. Currently, a solution is used, which involves converting the PAF (Potentially Affected Fraction) unit to PDF (Potentially Disappeared Fraction). In ecosystem monitoring processes, the degree of disturbance is the most important parameter. In the Eco-indicator 99 method, the disturbance indicator is the level of species diversity. Within individual impact categories, representative species groups were selected. For ecotoxicity (PAF·m^2^/yr.) it was lower species of terrestrial and aquatic animals, while levels of acidification/eutrophication and land use (PAF·m^2^/yr.) were referred to as selected species of vascular plants [26,40,41].

Modeling under the third area of influence—resources, consists of resource analysis and damage analysis. The Eco-indicator 99 considers only two impact categories from this area: minerals and fossil fuels. A special injury indicator was developed, analogous to DALY, PAF and PDF, which is surplus energy expressed in MJ. During resource analysis, the decline in the useful component content in the deposit or the depletion of the deposit is modeled (as potential mining effects). Damage is combined with additional energy necessary for the extraction of the resource in question in the future, which results from a reduction in supply (the effect of impoverishment or depletion of the deposit). The main assumption is based on the relationship that if the quality of a given resource decreases (due to an increase in its extraction), efforts to obtain it from other sources increase (surplus energy) [36,42,43].

Normalization is understood as the calculation of the size of the results of the category indicator relative to reference information. It is helpful when determining the relative importance of indicator results related to a given region (e.g., Europe), persons (e.g., average European resident) over a given period of time. Normalization can also be used to prepare LCIA results for subsequent procedures, e.g., weighting. The results of the indicator at the characterization stage are obtained in different units, so it would be difficult to assign them specific weighting factors and then multiply them. Conversion to a common unit by normalization allows for subsequent weighting [30].

There are different ways and preferences for valuation in relation to impact categories. Depending on the purpose and scope of the analysis, some may seem more important than others. In the Eco-indicator 99 method, grouping takes place when the results of impact categories are added to three areas of influence and during the final aggregation to Ecolabel [26,44,45,46].

Weighting consists of determining and assigning the importance level (weighting factor) to individual impact categories and multiplying them by normalized results of the indicator. Carrying out the weighting process allows results in environmental points (Pt) to be obtained. One thousand environmental points is equal to the impact on the environment of one European in one year [26].

#### 2.4.2. IPCC Method

The most important gases that increase the greenhouse effect include: CO_2_, N_2_O, CH_4_ and CFCs. When analyzing, it is important to take into account the residing time of a given gas in the atmosphere, because to estimate the greenhouse effect, it is important that the effects are long-lasting and have a global reach. In the IPCC (Intergovernmental Panel on Climate Change, Global Warming Potential) method, the greenhouse effect is measured by a value called GWP (Global Warming Potential). Carbon dioxide was chosen as the reference substance. For this reason, the results obtained are presented as kg CO_2_ eq per 1 mg (waste). The total indicator of the greenhouse effect impact related to CO_2_ is 1. The time horizon in the IPCC method is assumed for 20, 100 or 500 years. During the analyzes carried out as part of this study, 100 years were assumed as the period of consideration of the impact of various gases on the greenhouse effect [47,48,49,50].

#### 2.4.3. CED Method

As mentioned before, the Eco-indicator 99 method was used for ecological analyzes, while the CED method was used to determine the magnitude of the impact on the environment of processes related to obtaining energy in the lifecycle of the waste under consideration. The CED (Cumulative Energy Demand) method allowed the determination the cumulative energy demand. Impact indicators in this method are divided into seven impact categories: two categories relate to nonrenewable energy sources (fossil, nuclear), and five to renewable energy sources (biomass, wind, solar, geothermal, water). At the characterization stage, results are obtained in MJ, but thanks to normalization, grouping and weighting, as in Eco-indicator 99, results are also obtained in environmental points (Pt) [51,52,53].

### 2.5. Interpretation

During the analysis, its completeness was checked with a positive result. All key information and data necessary for interpretation were complete and obtained directly from the company producing wind power plant blades or were downloaded from the SimaPro 8.4 program databases. Compliance checks were also performed during the analysis. The adopted assumptions, methods used, depth of analysis, detail and precision of data for all considered postproduction waste of wind power plant blades, were consistent with the assumed goal and scope of research. The results of the analysis of differences for eight postproduction wastes and their detailed interpretation are presented in Section 3 and Section 4 [26,30].

## 3. Results of Environment and Technical System Control

The results of environment and system control analyzes carried out as part of the Life Cycle Impact Assessment (LCIA) are summarized in three sections including Eco-indicator 99 (Section 3.1), IPCC (Section 3.2) and CED (Section 3.3). Modeling results using Eco-indicator 99 and IPCC were divided into four areas of emission of individual chemical compounds: air, water, soil and raw.

All results were presented in units specific to the characterization stage per 1 mg—in the amount per one ton (megagram) of waste analyzed from the wind power plant blades production process [26,54].

### 3.1. Eco-Indicator 99

As part of research using Eco-indicator 99, a detailed analysis of eleven impact categories, characteristic for this model, was made: carcinogens, resp. organics, resp. inorganics, climate change, radiation, ozone layer, eco toxicity, acidification/eutrophication, land use, minerals and fossil fuels.

The results were compiled for the life cycle and for recycling processes of eight selected wastes from the wind power plant blades manufacturing process: fiberglass mat, roving fabric, resin discs, distribution hoses, spiral hoses with resin, vacuum bag film, infusion materials residues and surplus materials (their detailed characteristics are described in Section 2.3).

The first step of the analysis included assessing which of the eleven categories considered could be the source of potentially the largest number of negative (or positive) environmental consequences in the lifecycle and recycling processes of the tested waste.

Among the factors that may negatively affect human health, the highest level of harmful effects was characterized by a group of inorganic compounds causing respiratory diseases (from 1.17 × 10^−3^ DALY per 1 mg for vacuum bag film, to 3.64 × 10^−3^ DALY per 1 mg for roving fabric). During the production of wind power plant blades, significant amounts got into the environment, including nitrogen oxides and sulfur dioxide. The use of recycling processes for all analyzed wastes would result in a significant reduction in the adverse impact of the analyzed wastes in the considered impact category (from −3.94 × 10^−5^ DALY per 1 mg for vacuum bag film to −1.19 × 10^−3^ DALY per 1 mg for distribution hoses) in the perspective of the entire life cycle (Table 2 and Table 3).

In the group of factors affecting the reduction in environmental quality, two impact categories are of key importance—eco toxic substances (from 6.17 × 10^1^ PAF·m^2^/yr. per 1 mg for surplus materials to 9.55 × 10^2^ PAF·m^2^/yr. per 1 mg for fiberglass mat) and substances with an acidifying or eutrophic effect (from 3.51 × 10^1^PDF·m^2^/yr. per 1 mg for vacuum bag film to 9.88 × 10^1^ PDF·m^2^/yr. per 1 mg for resin discs). In the area of acidifying or eutrophic compounds, the use of recycling would reduce the negative impact over the entire lifecycle of all analyzed postproduction waste (from −3.19 × 10^0^ PDF·m^2^/yr. per 1 mg for vacuum bag film to −6.01 × 10^1^ PDF·m^2^/yr. per 1 mg for distribution hoses). Particularly high levels of reduction are achievable with one of the most environmentally hazardous substances—nitrogen oxides (Table 2 and Table 3).

Subsequently, among the factors associated with the depletion of fossil resources, the processes of fossil fuel extraction are by far the most detrimental (from 2.63 × 10^3^ MJ surplus per 1 mg for vacuum bag film to 3.41 × 10^4^ MJ surplus per 1 mg for resin discs). This is the result of high energy demand during the production of wind power plant blades and the associated energy-intensive processes of extracting nonrenewable raw materials (necessary during the production of blades). The use of recycling in the case of all waste considered would reduce the potential negative impact over the entire lifecycle (from −8.61 × 10^2^ MJ surplus per 1 mg for vacuum bag film to −8.61 × 10^3^ for fiberglass mat and resin discs) (Table 2 and Table 3).

Almost all chemical substances that accompany processes in the life cycle of postproduction of waste of wind power plant blades can cause specific environmental hazards. In fact, it is difficult to pinpoint exactly what part of a given substance is causing global warming, for example, and what is toxic to ecosystems or acidification. Therefore, in ecological and energy analyzes, for determining potential threats, it is assumed that a given substance has an impact on each impact category to which it can potentially contribute [26].

Therefore, in order to identify the life cycle areas of the analyzed wind power plant blades more precisely, which potentially can have the greatest negative (or positive) impact on the environment, a detailed analysis of substances and processes occurring within all impact categories was carried out, including emission areas (compartment: air, water, soil, raw).

#### 3.1.1. Carcinogens

**Figure 4:**
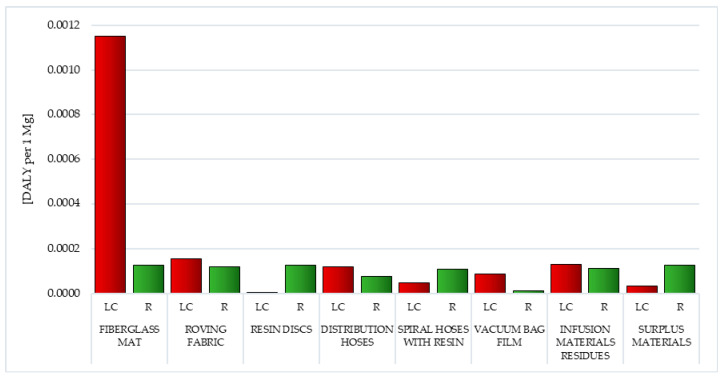
Characterization results of environmental consequences for carcinogens present in selected postproduction waste of wind power plant blades: LC—life cycle, R—recycling [unit: DALY per 1 mg].

Carcinogens are factors that through the mutation of genetic material contribute to the development of cancer. They are generated in the life cycle of all analyzed postproduction waste of wind power plant blades. The life cycle of fiberglass mat made of chopped glass fiber (total: 1.15 × 10^−3^ DALY per 1 mg) is particularly most dangerous in this respect, while the smallest—the life cycle of resin discs made of epoxy resin (total: 1.00 × 10^−6^ DALY per 1 mg) (Figure 4, Tables S1 and S2).

Among the carcinogens with the highest level of potential negative impact on human health, emitted into the atmosphere, cadmium can be distinguished (from 0.01 × 10^−9^ DALY per 1 mg for resin discs to 6.03 × 10^−4^ DALY per 1 mg for fiberglass mat), and among those emitted to the aquatic environment—arsenic ions (from 0.01 × 10^−9^ DALY per 1 mg for resin discs to 1.40 × 10^−4^ DALY per 1 mg for fiberglass mat). Cadmium belongs to the group of heavy metals and its introduction into the environment occurs both during production processes and during the storage of waste. Its excess in the body causes the formation of toxic effects, for example, damages the kidneys, causes bone or cardiovascular diseases. Cadmium is located, among others, in fossil fuels; it can, therefore, be released into the environment in the form of dusts generated during combustion (or in waste). The processes of producing, wind power plant blades are characterized by high energy consumption, and during the combustion of fuels, the most volatile elements, such as arsenic, also evaporate. At lower temperatures, these vapors condense, creating very small solid particles that are particularly harmful to humans. Arsenic may be the cause of, among others, liver or bronchus cancer (Tables S1 and S2) [55].

Postuse management in the form of recycling allows for a slight minimization of the negative impacts of the analyzed impact category in the perspective of the life cycle of the tested waste, especially in the case of metallic ion emissions (from −6.82 × 10^−7^ DALY per 1 mg for vacuum bag film to −6.82 × 10^−6^ DALY per 1 mg for fiberglass mat and resin discs). However, overall, the recycling of waste generated during the production of wind power plant blades is also associated with emissions of carcinogens into the environment (including those arising during chemical reactions that occur as a result of the use of reagents in such processes). Fiberglass mat and resin discs (total: 1.27 × 10^−4^ DALY per 1 mg) have the highest level of harmful effect in this area, and the lowest—vacuum bag film (total: 1.27 × 10^−5^ DALY per 1 mg) (Figure 4, Tables S1 and S2).

#### 3.1.2. Organic Compounds Causing Respiratory Diseases

**Figure 5:**
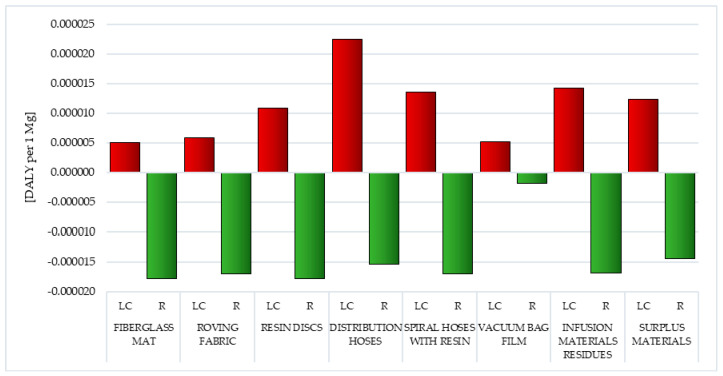
Characterization results of environmental consequences for organic compounds causing respiratory diseases present in selected postproduction waste of wind power plant blades: LC—life cycle, R—recycling [unit: DALY per 1 mg].

Respiratory diseases affect an increasing proportion of the world’s population. The most common include infectious diseases (for example: cold, pneumonia), cancer (such as lung cancer), occupational (such as pneumoconiosis), genetic (for example: cystic fibrosis), but also, a number of other conditions such as allergies. Some of these can be caused by both organic and inorganic compounds arising in the life cycle of postproduction waste of wind power plant blades. The largest source of emission of organic compounds causing respiratory diseases among all analyzed wastes was the life cycle of distribution hoses (inner and outer layer made of high quality PVC material, reinforcement—polyester fiber)—total: 2.25 × 10^−5^ DALY per 1 mg. The least emissions under this impact category were recorded for the vacuum bag film life cycle (made of co-extrusion of polyolefin and nylon based resins)—total: 5.17 × 10^−6^ DALY per 1 per 1 mg (Figure 5, Tables S3 and S4) [55].

For the majority of postproduction waste considered, emissions of nonmethane volatile organic compounds had the greatest impact on the overall level of negative impacts (from 0.02 × 10^−9^ DALY per 1 mg for resin discs to 1.24 × 10^−5^ DALY per 1 mg for spiral hoses with resin) that easily turn into steam or gas. They occur as byproducts in many industrial processes, including during the production of wind power plant blades and are a source of environmental pollution and they also have a carcinogenic effect. An exception in the analyzed impact category was resin discs and distribution hoses. Their total harmful effect is mainly due to emissions of some hydrocarbons, which are the basic component of crude oil (in sequence 1.07 × 10^−5^ and 2.19 × 10^−5^ DALY per 1 mg), which is necessary in the production of most polymer materials. Their emissions may include an increase in the incidence of asthma (Tables S3 and S4) [55].

Recycling processes would greatly reduce the level of negative impact of the considered compounds in the perspective of the entire life cycle of tested postproduction waste. The highest reduction level, totally −1.77 × 10^−5^ DALY per 1 mg, was recorded for fiberglass mat and resin discs. These results primarily consisted of limiting the harmful effects of nonmethane volatile organic compound emissions (from −1.79 × 10^−6^ DALY per 1 mg for vacuum bag film to −1.79 × 10^−5^ DALY per 1 mg for fiberglass mat and resin discs) (Figure 5, Tables S3 and S4).

#### 3.1.3. Inorganic Compounds Causing Respiratory Diseases

**Figure 6:**
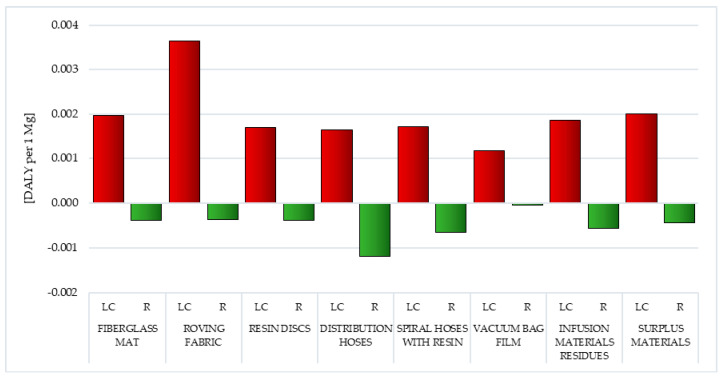
Characterization results of environmental consequences for inorganic compounds causing respiratory diseases present in selected postproduction waste of wind power plant blades: LC—life cycle, R—recycling [unit: DALY per 1 mg].

The impact category characterized by the highest level of potential harmful effects on human health was the emission of inorganic compounds that cause respiratory diseases. The greatest threat to human and animal health in this area was the cycles of existence of waste generated mainly from fiberglass, namely fiberglass mat (total: 1.96 × 10^−3^ DALY per 1 mg) and roving fabric (total: 3.64 × 10^−3^ DALY per 1 mg), while the smallest was the vacuum bag film life cycle (total: 1.17 × 10^−3^ DALY per 1 mg) (Figure 6, Tables S5 and S6).

The total size of potential negative impacts in almost all analyzed postproduction waste of wind power plant blades was determined by the level of nitrogen oxide emission (from 3.71 × 10^−4^ DALY per 1 mg for vacuum bag film to 1.51 × 10^−3^ DALY per 1 mg for resin discs). The only exception was the vacuum bag film, where sulfur oxides (5.86 × 10^−4^ DALY per 1 mg) played the most important role. Nitrogen oxide is formed mainly in the processes of burning fossil fuels as a result of secondary processes. The intensity of its creation affects the course of many different chemical mechanisms, depending on the type of fuel, its composition or combustion conditions. It contributes to the formation of chronic bronchitis, emphysema and increases the susceptibility to respiratory infections. On the other hand, sulfur oxide emissions are the result of the presence of sulfur compounds in fuels (for example in coal, sulfur occurs mainly in the form of FeS_2_ pyrites, and in crude oil—in the form of organic compounds). Sulfur oxides may react with water vapor or water droplets to form sulfuric acid or with dusts (often containing metal oxides) to form sulfates. Sulfur oxides strongly irritate the respiratory tract and have harmful effects on plant growth and development. As mentioned before, the production processes of wind power plant blades are distinguished by a large demand for energy, which today still comes mainly from conventional sources (Tables S5 and S6) [55].

Recycling of the researched postproduction waste can significantly reduce its potential negative impact on the environment over the entire life cycle. The largest reduction in this type of processes was in the case of distribution hoses (total: −1.19 × 10^−3^ DALY per 1 mg). For all wind power plant blades, the magnitude of positive impacts in this area was mainly due to the reduction in the harmful effects of nitrogen oxide emissions (from −98 × 10^−4^ DALY per 1 mg for surplus materials to −8.93 × 10^−4^ DALY per 1 mg for distribution hoses) (Figure 6, Tables S5 and S6).

#### 3.1.4. Climate Change

**Figure 7:**
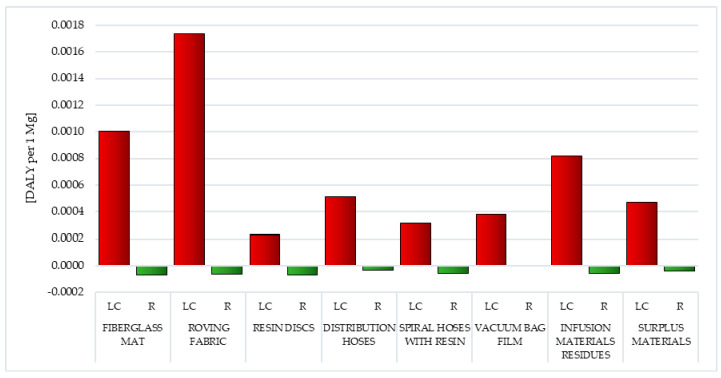
Characterization results of environmental consequences for compounds causing climate change present in selected postproduction waste of wind power plant blades: LC—life cycle, R—recycling [unit: DALY per 1 mg].

The Earth’s climate is primarily influenced by the temperature of air, water, soil surface, precipitation and solar radiation. The natural greenhouse effect in the atmosphere is caused by the following (in small quantities): water vapor, carbon dioxide, tropospheric ozone, nitrous oxide, methane, aerosols, cloud particles and other trace gases. CO_2_, whose key anthropogenic sources are fossil fuel combustion and deforestation, has the largest share in the expansion of the greenhouse effect. CH_4_ emissions (fossil fuels, waste, farming, rice cultivation), freons (aerosols, foams) and nitrous oxides (eutrophication, deforestation, biomass burning) are also important. A rise in temperature at the Earth’s surface due to excessive greenhouse effects can lead to severe climate change. For example, decreasing the thickness of the ice cover at the poles can cause ocean water levels to rise and changes in rainfall zones, which, in turn, can shift vegetation zones. The largest harmful effects on the environment of compounds causing climate change were recorded for the life cycle of roving fabric (total: 1.74 × 10^−3^ DALY per 1 mg) and fiberglass mat (total: 1.01 × 10^−3^ DALY per 1 mg), which are formed mainly from fiberglass. The production of fiberglass is associated with the need to incur large energy expenditure, which is usually met by burning conventional fuels. This results in the depletion of nonrenewable resources as well as the reduction in environmental quality and the creation of a number of health problems. The smallest impact in the area of climate change was characterized by the life cycle of resin discs (total: 2.32 × 10^−4^ DALY per 1 mg) made of epoxy resin (Figure 7, Tables S7 and S8) [56].

Carbon dioxide emissions are the most important element affecting the total impact of all tested wind power plant blades, as part of the analyzed impact category. The use of recycling would reduce the adverse effects of compounds that cause climate change (especially carbon dioxide) over the entire life cycle. The highest level of total reduction was characterized by fiberglass mat and resin discs (total: −6.95 × 10^−5^ DALY per 1 mg), while the lowest was vacuum bag film (total: −6.95 × 10^−6^ DALY per 1 mg) (Figure 7, Tables S7 and S8) [56].

#### 3.1.5. Radiation

The source of radiation is the distribution of some elements found in nature or artificial elements, known as radionuclides. Radioactive contamination can be caused not only by the production and processing of fuels for nuclear reactors but also (although to a lesser extent, of course) by the exploitation, storage and processing of minerals—hard coal or copper ores. The production of wind power plant blades and related processes of extracting raw materials are associated with the demand for large amounts of materials and energy. Energy for this purpose is obtained mainly from nonrenewable sources such as coal. Radioactive elements include, for example, thorium, uranium and their degradation products, such as radon or radium. In chemical terms, these elements are less toxic than some other carbon components (namely, mercury, selenium, arsenic), but they can pose a threat to human health, precisely, because of their radioactive properties [57].

**Figure 8:**
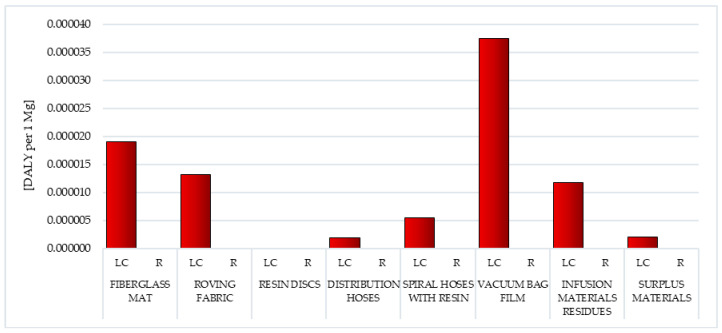
Characterization results of environmental consequences for radioactive substances present in selected postproduction waste of wind power plant blades: LC—life cycle, R—recycling [unit: DALY per 1 mg].

Vacuum bag film was postproduction waste characterized by the highest level of emission of hazardous radioactive substances in the life cycle (total: 3.74 × 10^−5^ DALY per 1 mg), while the lowest was characterized by the cycles of existence of distribution hoses (total: 1.91 × 10^−6^ DALY per 1 mg) and surplus materials (total: 2.10 × 10^−6^ DALY per 1 mg). The size of potential impacts under this impact category was mainly due to the ^222^Radon isotope emissions recorded in the lifecycle of almost all waste considered wind power plant blades (from 1.29 × 10^−6^ DALY per 1 mg for distribution hoses to 3.17 × 10^−5^ DALY per 1 mg for vacuum bag film) (Figure 8, Tables S9 and S10).

#### 3.1.6. Ozone Layer

**Figure 9:**
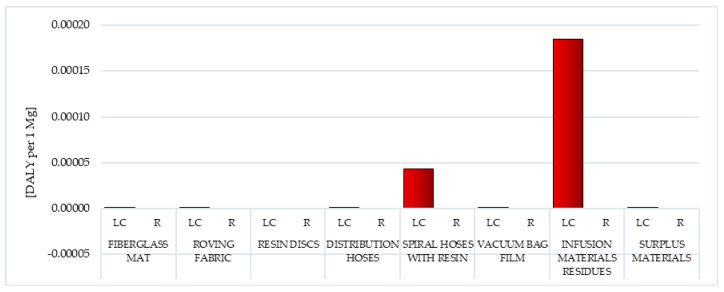
Characterization results of environmental consequences for compounds causing an increase in the ozone hole present in selected postproduction waste of wind power plant blades: LC—life cycle, R—recycling [unit: DALY per 1 mg].

Anthropogenic emissions can cause depletion of the ozone in the stratosphere. Systematic observations carried out since the early seventies have shown that the thickness of the ozone layer is reduced. Its ongoing degradation can have serious consequences for the environment. Among the effects is, for example, greater heating of the Earth’s surface, and consequently, a reduction in harvest (due to photosynthesis disorders), an increase in skin cancers, diseases of the eye and a reduction in the amount of plankton in the seas and oceans, which is a key link in many food chains. The highest emission level of compounds causing the ozone hole enlargement was recorded for the life cycle of infusion materials residues (total: 1.85 × 10^−4^ DALY per 1 mg) and spiral hoses with resin (total: 4.30 × 10^−5^ DALY per 1 mg), while the lowest was for resin discs (total: 0.02 × 10^−9^ DALY per 1 mg) (Figure 9, Tables S11 and S12) [58].

The most important factors shaping the magnitude of this type of impact are emissions of tetrachloromethane (CFC-10), bromotrifluoromethane (Halon 1301) and dichlorodifluoromethane (CFC-12). They not only destroy the ozone layer but also show direct harm to human health. They cause, among others, diseases of the nervous system, liver or kidneys. They are sometimes used as solvents in some chemical reactions, but successive steps are taken to stop using them. The use of recycling processes can, to some extent, reduce the negative impact of compounds that cause the ozone hole to expand, especially in the area of bromotrifluoromethane (Halon 1301) emissions (from −4.74 × 10^−8^ DALY per 1 mg for vacuum bag film to −5.43 × 10^−7^ DALY per 1 mg for surplus materials). The highest level of total reduction in harmful effects was recorded for fiberglass mat and resin discs (total: −4.74 × 10^−7^ DALY per 1 mg) (Figure 9, Tables S11 and S12) [58].

#### 3.1.7. Ecotoxicity

**Figure 10:**
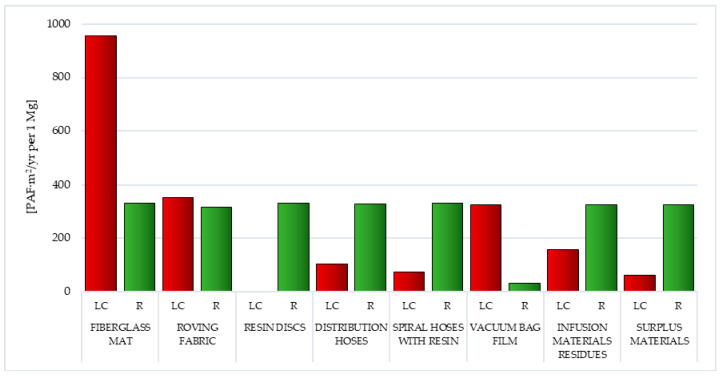
Characterization results of environmental consequences for ecotoxic compounds present in selected postproduction waste of wind power plant blades: LC—life cycle, R—recycling [unit: PAF·m^2^/yr per 1 mg].

Ecotoxicity is a feature of some substances that causes (or potentially can cause) immediate (or delayed) risk for one (or more) elements of the environment. Substances of this type can, therefore, significantly reduce the quality of the environment, and as a consequence, pose a threat to human and animal health. Of all the types of waste generated during the production of wind power plant blades, the most ecotoxic emissions were recorded for the fiberglass mat life cycle made of chopped glass fiber (total: 9.55 × 10^2^ PAF·m^2^/yr. per 1 mg while the least was for the life cycle resin discs made of epoxy resin (total: 6.96 × 10^−2^ PAF·m^2^/yr. per 1 mg) (Figure 10, Tables S13 and S14) [55].

The total environmental impact was mainly shaped by cadmium emissions (from 0.01 × 10^−9^ PAF·m^2^/yr. per 1 mg for resin discs to 4.31 × 10^2^ PAF·m^2^/yr. per 1 mg for fiberglass mat), related to blade production processes. Additionally, in fossil fuels (such as hard coal) there are significantly large amounts of it. Therefore, due to their extraction and subsequent processing, it is emitted to the environment. Cadmium belongs to the group of heavy metals and is one of the more dangerous environmental pollutants. This element is found in the air most often in the form of oxides that are effectively soluble in water. Therefore, it is very easy to contaminate aquatic ecosystems but also soil. It is particularly dangerous due to its rapid absorption by living organisms and easy accumulation in plant and animal tissues. The most common effects of this element include liver, kidney, lung damage, osteoporosis, anemia and cancer. Cadmium also contributes to changes in the functionality of cell membranes, inhibiting cell division and reducing the efficiency of photosynthesis (Tables S13 and S14) [55,57].

Wind power plant blades waste recycling is associated with emissions of ecotoxic substances (including those created during chemical reactions that occur as a result of the use of various types of reagents and the need to provide energy that comes mainly from conventional fuels). The least harmful emissions in this area were recorded for vacuum bag film (total: 3.32 × 10^1^ PAF·m^2^/yr. per 1 mg), while for other analyzed wastes, their size was similar and ranged from 3.15 × 10^2^ PAF·m^2^/yr. per 1 mg for roving fabric to 3.32 × 10^2^ PAF·m^2^/yr. per 1 mg for fiberglass mat and resin discs (Figure 10, Tables S13 and S14).

#### 3.1.8. Acidification/Eutrophication

Soil and surface water are acidified mainly by converting air pollutants into acids. In the case of postproduction waste of wind power plant blades, most often, nitrogen and sulfur oxides are hydrated in the atmosphere and fall to the Earth’s surface in the form of acids contained in rain, snow and fog or in the form of dry particles. The presence of acids in the environment is the cause of the leaching of nutrients and increasing the solubility of metals in the soil. This destroys ecosystems and leads to the acidification of surface waters. In acidic waters, the destruction of living organisms occurs, mainly as a result of the dissolution of heavy metals. Soil acidification in turn causes the disturbance of vegetation processes (Tables S15 and S16) [55].

The term eutrophication means the process of enriching with nutrients. Visible symptoms of excessive eutrophication are: intensive growth and development of aquatic plant, a large amount of silt, water turbidity, its unpleasant taste and smell. The factor accelerating water degradation is increased anthropogenic activity, including impacts related to the production of blades. The indicators of the development of the eutrophication process are: a decrease in the oxygen concentration in water, an increase in the concentration of various forms of nitrogen and phosphorus as well as a significant increase in the amount of biomass. Reducing the concentration of oxygen in water leads to the dying of fish and anaerobic degradation, which results in, among others, methane and sulfur oxides. This leads to damaging ecosystems (Tables S15 and S16) [55].

**Figure 11:**
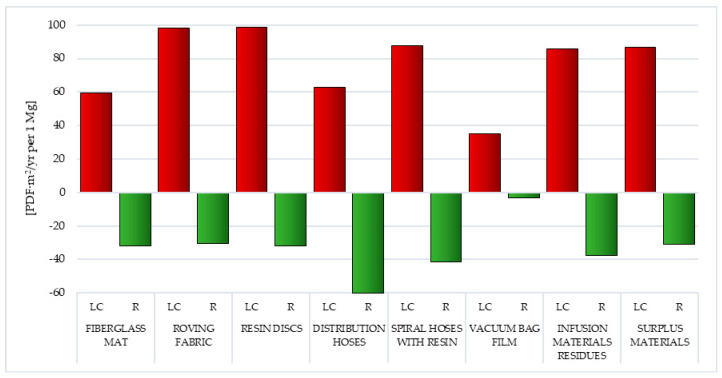
Characterization results of environmental consequences for compounds causing acidification or eutrophication present in selected postproduction waste of wind power plant blades: LC—life cycle, R—recycling [unit: PDF·m^2^/yr per 1 mg].

The life cycle of resin discs made of epoxy resin (total: 9.88 × 10^1^ PDF·m^2^/yr. per 1 mg) and roving fabric made of epoxy-silane coated fiberglass had the greatest impact on the reduction in environmental quality, caused by the emission of acidifying or eutrophic compounds. (total: 9.84 × 10^1^ PDF·m^2^/yr. per 1 mg). The lowest level of negative influences under this impact category was the vacuum bag film life cycle made of co-extrusion of polyolefin and nylon based resins (total: 3.51 × 10^1^ PDF·m^2^/yr. per 1 mg). The emission of the aforementioned nitrogen oxides (from 9.69 × 10^1^ PDF·m^2^/yr. per 1 mg for resin discs to 2.39 × 10^1^ PDF·m^2^/yr. per 1 mg for vacuum bag film) was of key importance during the shaping of these quantities (Figure 11, Tables S15 and S16).

Recycling of postproduction waste of wind power plant blades can contribute to lowering the level of harmful effects of compounds causing acidification or eutrophication in the perspective of their entire life cycle. The highest degree of potential reduction was observed in the case of distribution hoses made of high quality PVC and polyester fiber (total: −6.01 × 10^1^ PDF·m^2^/yr. per 1 mg), while the lowest was for vacuum bag film (total: −3.19 × 10^0^ PDF·m^2^/yr. per 1 mg). In this area, the most important was limiting the impact of nitrogen oxide emissions (from −3.44 ×·10^0^ PDF·m^2^/yr. per 1 mg for vacuum bag film to −5.75 × 10^1^ PDF·m^2^/yr. per 1 mg for distribution hoses) (Figure 11, Tables S15 and S16).

#### 3.1.9. Land Use

Exploitation of natural resources of the environment leads to their depletion and even complete depletion. The most protected resources at risk of degradation or depletion include: fossil fuel resources, nonenergy mineral resources, potable and industrial water resources, land and soil resources. In relation to lands and soils, the concept of devastation is distinguished when the land has completely lost its agricultural or forestry value in use and the concept of degradation—when there is a deterioration in utility properties, deformation of biological activity by changing the reaction (acidification, alkalization, content of trace elements, especially metals) and also microbial activity. Economic activities, including those related to the production of wind power plant blades, lead to soil transformations of a geomechanical, hydrological type and degradation as a result of chemical pollution [57].

**Figure 12:**
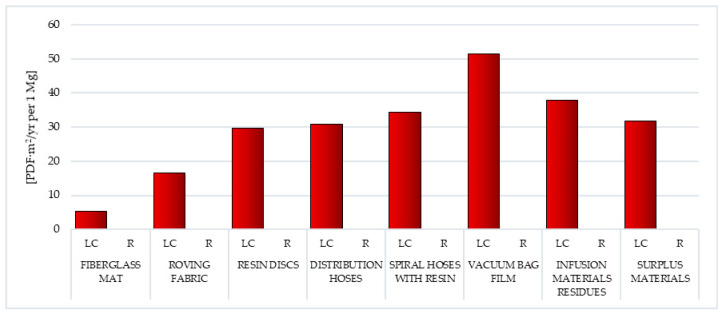
Characterization results of environmental consequences for processes related to land use, present in selected postproduction waste of wind power plant blades: LC—life cycle, R—recycling [unit: PDF·m^2^/yr per 1 mg].

Of all the types of waste analyzed, the highest level of harmful impact caused by land use processes was characterized by the vacuum bag film life cycle (total: 5.15 × 10^1^ PDF·m^2^/yr. per 1 mg), while the smallest was fiberglass mat (total: 5.32 × 10^0^ PDF·m^2^/yr. per 1 mg). The size of the total impact under this impact category, for most wastes, was the process of occupation of industrial area (from 2.50 × 10^−1^ PDF·m^2^/yr. per 1 mg for roving fabric to 3.03 × 10^1^ PDF·m^2^/yr. per 1 mg for distribution hoses). The exceptions were the fiberglass mat life cycle (the highest value was recorded for process transformation to mineral extraction site), roving fabric (process occupation forest intensive or normal) and vacuum bag film (process land use—class II-III) (Figure 12, Tables S17 and S18).

#### 3.1.10. Minerals

Each exploitation of minerals (solid, liquid and gas) causes not only depletion of resources but also a violation of the current state of the environment, expressed in its transformation and deterioration of quality. In the case of obtaining raw materials, changes in the environment largely depend on the mining methods (for example underground, opencast, borehole). As a result of underground mining exploitation, there are displacements of rock mass elements that cause deformation of the land surface. These movements can also cause changes in water relations in the rock mass and on the surface. Mining operations may also be associated with the formation of various seismic phenomena. On the other hand, in surface mining, raw materials are removed and stored near the excavation, where they are exposed to erosion and chemical weathering, leaching or washing out. The impact of mining opencast works on the environment consists mainly in the transformation or long-term decommissioning of soils, violation of the construction and change in the physical and mechanical features of the rock mass, transformation of terrain morphology, qualitative and quantitative changes in surface and deep waters, violation of landscape values and deterioration of recreational values of the region. In some cases, soil desiccation and reduction in crops as well as other forms of mining damage occur (damage to buildings, roads, waterways, power lines, etc.). In addition, emerging external dumps strongly interfere with the topography. The raw materials used in the production of polymer materials are usually organic materials and natural raw materials, for example: cellulose, coal, natural gas and crude oil. However, in the processes of producing wind power plant blades, it is also necessary to provide a certain amount of mineral resources, most often obtained in open-cast mines [57].

**Figure 13:**
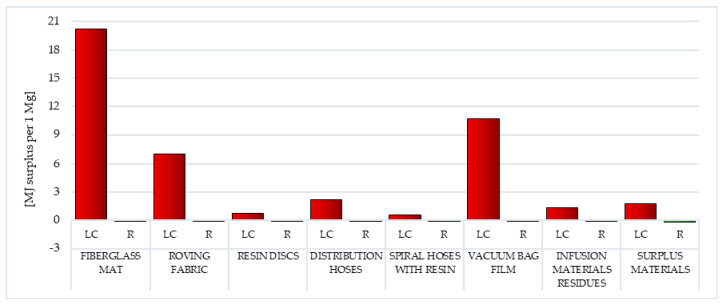
Characterization results of environmental consequences for processes related to the extraction of mineral resources present in selected postproduction waste of wind power plant blades: LC—life cycle, R—recycling [unit: MJ surplus per 1 mg].

The most potential negative environmental consequence associated with the extraction of mineral resources was distinguished by the fiberglass mat life cycle (total: 2.02 × 10^1^ MJ surplus per 1 mg), while the least was the cycle of the existence of spiral hoses with resin (total: 5.96 × 10^−1^ MJ surplus per 1 mg). The largest number of harmful impacts can potentially be caused by processes related to the acquisition and production of aluminum, which begins with the construction of the bauxite opencast mine (up to a maximum of 1.94 × 10^1^ MJ surplus per 1 mg for fiberglass mat). Additionally, copper (up to a maximum of 9.38 × 10^0^ MJ surplus per 1 mg for vacuum bag film) and tin (up to a maximum of 2.60 × 10^0^ MJ surplus per 1 mg for roving fabric) are most often mined in this type of mine (Figure 13, Tables S19 and S20).

The use of recycling processes may slightly reduce the potential adverse impacts under this impact category. The highest total reduction level was characterized by surplus materials (total: −1.57 × 10^−1^ MJ surplus per 1 mg), while the lowest was vacuum bag film (total: −1.40 × 10^−2^ MJ surplus per 1 mg). Its size primarily shaped the potential for reducing the harmful effects of bauxite mining processes (from −1.35 × 10^−2^ MJ surplus per 1 mg for vacuum bag film to −1.51 × 10^−1^ MJ surplus per 1 mg for surplus materials) (Figure 13, Tables S19 and S20).

#### 3.1.11. Fossil Fuels

**Figure 14:**
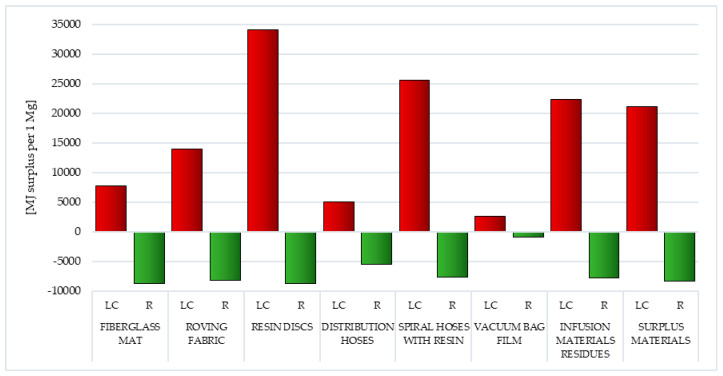
Characterization results of environmental consequences for processes related to the extraction of fossil fuels present in selected postproduction waste of wind power plant blades: LC—life cycle, R—recycling [unit: MJ surplus per 1 mg].

As already mentioned in the previous section, fossil fuels, most often used for the production of polymer materials, include crude oil, coal and natural gas. Processes related to their extraction (and combustion for energy purposes) cause a significant reduction in the quality of the environment. The production of wind power plant blades requires a lot of energy and matter. Particularly high levels of potential negative impacts were recorded in the life cycle of resin discs made of epoxy resin (total: 3.41 × 10^4^ MJ surplus per 1 mg) and extremely low levels for vacuum bag film made of co-extrusion of polyolefin and nylon based resins (total: 2.63 × 10^3^ MJ surplus per 1 mg). The total level of harmful environmental consequences in the case of waste produced mainly from fiberglass (fiberglass mat and roving fabric), was mainly composed of the impact of processes related to natural gas extraction (in sequence 4.60 × 10^3^ and 8.81 × 10^3^ MJ surplus per 1 mg). For the remaining types of postproduction waste, it was primarily the impact of processes related to obtaining crude oil (maximally 3.10 × 10^4^ MJ surplus per 1 mg for resin discs) (Figure 14, Tables S21 and S22).

Recycling can reduce the amount of harmful effects in an area. The best results were recorded in the case of cycles of existence of fiberglass mat and resin discs (total: −8.61 × 10^3^ MJ surplus per 1 mg), while the lowest level of reduction was for vacuum bag film (total: −8.61 × 10^2^ MJ surplus per 1 mg). Their level was shaped mainly by the possibilities of potential reduction in the negative impact of processes related to crude oil extraction (maximally −9.25 × 10^2^ MJ surplus per 1 mg for surplus materials) (Figure 14, Tables S21 and S22).

### 3.2. IPCC Method

In the modern world, public opinion is paying more and more attention to deepening environmental problems, in particular, aspects related to global warming. For this reason, additional analyses were carried out using the IPCC method, aimed at more accurately assessing the volume of individual greenhouse gas emissions over the life cycle and during the recycling of the eight considered postproduction waste of wind power plant blades. The obtained results were presented in the unit of kg CO_2_ eq per 1 mg.

**Figure 15:**
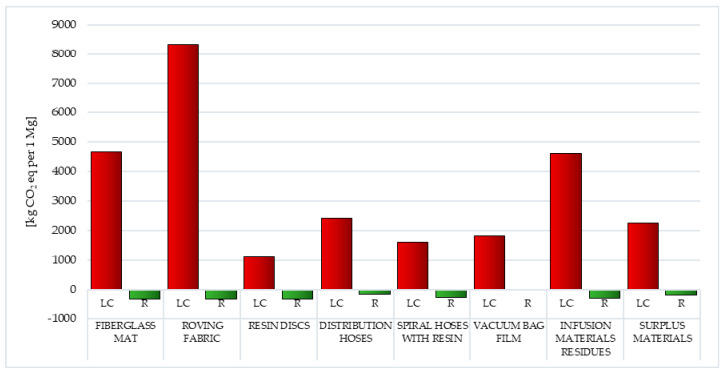
Characterization results of environmental consequences for processes related to greenhouse gas emissions (GHG) for selected postproduction waste of wind power plant blades: LC—life cycle, R—recycling [unit: kg CO_2_ eq per 1 mg].

Carbon dioxide comes mainly from the burning of fossil fuels. The amount of CO_2_ generated depends, among other things, on the type of fuel burned. The lowest carbon content among conventional fuels is characterized by natural gas, the main component of which is methane. The fuels with the highest amount of carbon are (most often used) hard coal and lignite, which have very high carbon dioxide emission factors. The residence time of carbon dioxide in the atmosphere is estimated at 50–200 years. In turn, most of the carbon monoxide (CO) in the air comes from CH_4_ oxidation processes. Carbon monoxide is formed in combustion processes that occur with oxygen deficiency or insufficient mixing of fuel and air. The highest level of greenhouse gas emissions (GHG) was characterized by the life cycle of postproduction waste of wind power plant blades, in which the largest mass share was fiberglass—i.e., fiberglass mat and roving fabric (in sequence 4.68 × 10^3^ and 8.33 × 10^3^ kg CO_2_ eq per 1 mg), while the lowest was life cycle of resin discs made of epoxy resin (total: 1.11 × 10^3^ kg CO_2_ eq per 1 mg) (Figure 15, Table 4 and Table 5) [57].

Carbon dioxide (maximally 7.35 × 10^3^ kg CO_2_ eq per 1 mg for roving fabric) is a key substance that increases the greenhouse effect in the perspective of the life cycle of the analyzed waste. The only exceptions are infusion material residues, where this role is performed by chlorodifluoromethane (HCFC-22)—1.69 × 10^3^ kg CO_2_ eq per 1 mg. Under conditions of elevated temperature, toxic decomposition products may be formed, such as: hydrogen fluoride, hydrogen chloride, carbon monoxide, carbon dioxide or chlorine, posing a threat to both human health and causing a decrease in the quality of the environment (Figure 15, Table 4 and Table 5) [55].

Postuse management in the form of recycling can, to some extent, reduce the negative environmental consequences associated with greenhouse gas emissions, especially in the case of carbon dioxide emissions (maximally −3.37 × 10^2^ kg CO_2_ eq per 1 mg for fiberglass mat and resin discs). The highest reduction in harmful emissions was characterized by the life cycle of fiberglass mat and resin discs (total: −3.33 × 10^2^ kg CO_2_ eq per 1 mg), the lowest was vacuum bag film (total: −3.33 × 10^1^ kg CO_2_ eq per 1 mg) (Figure 15, Table 4 and Table 5).

### 3.3. CED Method

As mentioned before, the results of the research carried out under Life Cycle Impact Assessment (LCIA) were compiled in three sections—the first of them included the Eco-Indicator 99 method (Section 3.1), the second—the IPCC method (Section 3.2), and the third—the CED method (Section 3.3). The results obtained as part of the characterization stage by the CED method were presented in the unit MJ eq per 1 mg. Similarly to the previous sections, the results obtained were compiled separately for the life cycle and for postuse management processes in the form of recycling

**Figure 16:**
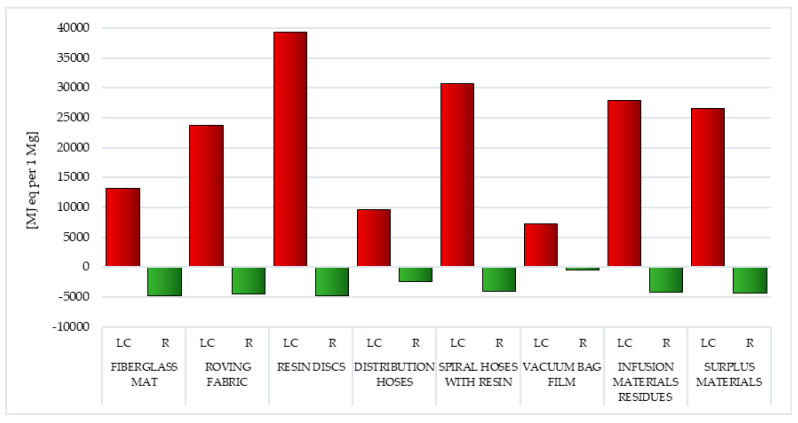
Characterization results of environmental consequences for processes related to energy production for selected postproduction waste of wind power plant blades: LC—life cycle, R—recycling [unit: MJ eq per 1 mg].

The main factor affecting the volume of emissions of substances hazardous to human health, chemicals that reduce the quality of the environment and the depletion of raw material resources is the need for energy. In the lifecycle of postproduction waste of wind power plant blades, the largest amount of energy was obtained from nonrenewable, fossil sources (from 4.74 × 10^3^ MJ eq per 1 mg for vacuum bag film to 3.93 × 10^4^ MJ eq per 1 mg for resin discs). Waste lifecycle was characterized by the highest energy consumption, in which the largest mass percentage was epoxy resin—resin discs and spiral hoses with resin (in sequence 3.93 × 10^4^ and 3.08 × 10^4^ MJ eq per 1 mg). The least energy was consumed in the life cycle of the vacuum bag film (total: 7.24 × 10^3^ MJ eq per 1 mg) (Figure 16, Table 6 and Table 7).

Recycling allows for limiting the negative consequences associated with energy production in the perspective of the entire life cycle of all types of postproduction waste tested. The highest reduction level was recorded for fiberglass mat and resin discs (total: −4.74 × 10^3^ MJ eq per 1 mg), and the lowest for vacuum bag film (total: −4.74 × 10^2^ MJ eq per 1 mg). Reducing the harmful effects of energy production from nonrenewable, fossil sources (from −8.31 × 10^2^ MJ eq per 1 mg for vacuum bag film to −8.31 × 10^3^ MJ eq per 1 mg for fiberglass mat and resin discs) was particularly important (Figure 16, Table 6 and Table 7).

## 4. Conclusions

Renewable energy is seen as the future of energy and is hoped to limit climate change. Although, so far, energy is mainly obtained from fossil fuels, public opinion is increasingly noticing that such a method of generating energy is the cause of environmental pollution, an increase in the average global temperature and the depletion of natural resources [59,60].

Wind energy is one of the branches of renewable energy. Although virtually no harmful chemicals are emitted to the environment during the operation of wind power plants, postuse management after the end of their operation is associated with a significant burden on the environment. Today, most problems occur during postuse management of wind power plant blades, mainly made of polymer materials [61,62].

The main goal of this analysis was achieved thanks to ecological and energetic environment and system control analysis of the life cycle of selected postproduction waste of wind power plant blades.

The environment and system control analyses were carried out based on the Life Cycle Assessment (LCA) method. Three calculation procedures were used: Eco-indicator 99, IPCC and CED. The results were compiled individually for eight wind power plant blades postuse wastes (fiberglass mat, roving fabric, resin discs, distribution hoses, spiral hoses with resin, vacuum bag film, infusion materials residues, surplus materials). The analysis covered almost the entire life cycles of the considered postproduction waste of wind power plant blades (it did not include the stages of waste storage and transport to postuse management).

Among the factors harmful to human health, the highest levels of negative impact were distinguished by inorganic compounds causing respiratory diseases. The greatest threat in this area is the cycles of existence of waste mainly produced from fiberglass (fiberglass mat and roving fabric). The total size of potential negative impacts is mainly determined by the level of nitrogen oxide emissions (Figure 6, Table 2 and Table 3).

In the group of factors that reduce the quality of the environment, ecotoxic substances and chemical compounds with acidifying or eutrophic action are of the greatest importance. Of all the types of waste tested, the most emissions of eco toxic compounds are emitted over the life cycle of fiberglass mats. The total environmental impact was in this case shaped primarily by cadmium emissions. Subsequently, the largest emissions of acidifying or eutrophic compounds were recorded for the life cycle of resin discs made of epoxy resin and roving fabric. The emission of the aforementioned nitrogen oxides is of key importance during shaping these quantities (Figure 10 and Figure 11, Table 2 and Table 3).

Within the factors related to resource depletion, the maximum negative impact characterizes the processes of extracting fossil fuels. The production of wind power plant blades requires considerable energy and matter. The particularly high level of potential negative influences in this area characterized the life cycle of resin discs. The total level of harmful environmental consequences under this impact category mainly consisted of the impact of processes related to crude oil extraction (Figure 14, Table 2 and Table 3).

The environmental impact associated with the extraction of minerals is primarily the degradation of the land, consisting of soil and rock displacement, loss of soil resources, destruction of vegetation, soil erosion, landfill movements, land subsidence due to exploitation and causing slope instability. As a result of these types of activities, groundwater degradation, surface water pollution by waste and chemicals, sedimentation of pollution in rivers and reservoirs, accumulation of large amounts of waste and water pollution caused by chemical pollution occur. In addition, the generated waste is usually stored in heaps near the extraction and processing site, interfering with the natural relief of the area and posing a threat to the environment [63].

Among the world’s most-known environmental problems is greenhouse gas (GHG) emissions. The highest level of their emission is distinguished by the life cycle of waste, in which the main mass share is fiberglass, for example: fiberglass mat and roving fabric. Carbon dioxide is the key substance that increases the greenhouse effect in the case of the analyzed waste (Figure 15).

The processes of extraction of raw materials, their processing and subsequent production of wind power plant blades are associated with the use of energy obtained mainly from conventional sources. This affects both the level of negative impact on the environment of their life cycle and depletion of raw material resources. The resin disc life cycle includes the most energy-consuming processes compared to other assessed waste, as a result of which, it also has the greatest destructive impact on the environment (Figure 16, Table 2 and Table 3).

There is a need to change the way environmental resources are managed, and experience to date shows the great opportunities that exist in rationalizing the use of nature resources. This requires creating more balanced attitudes in the sphere of production but also in all economic activity. It is necessary to fundamentally reorganize the entire economy in such a way as to enable the free flow of recyclable materials between recycling producers and companies and other industries [64,65,66].

The use of recycling processes can reduce the level of negative impact over the entire life cycle of all types of waste of wind power plant blades.

Among the activities for sustainable development is the improvement of already known manufacturing techniques and the search for new technologies. That is why the concept of best available technique (BAT) is particularly important, as it has become an important tool in this regard. It includes environmentally friendly methods of production, design, construction, operation and postuse management, which can be the basis for setting emission standards to prevent the progressive degradation of the environment [67,68,69].

When designing or improving the production processes of wind power plant blades, particular attention should be paid to:

1. The use of low-waste technology or (if possible) nonwaste technology;
2. Using substances with the lowest toxicity;
3. Increasing the level of recovery and reuse of substances used in individual technological processes;
4. Increasing the level of recovery and re-use of generated waste;
5. Following technological and scientific development;
6. Identifying the type and reduction in the amount and extent of emissions of pollutants into the environment;
7. Reducing the consumption of natural resources (including water);
8. Increasing energy and material efficiency of production processes;
9. The possibility of starting procedures related to the introduction of the best available technique.

Best Available Technique can be considered as an assessment of the progress of technological processes based on the emission of pollutants and the consumption of resources [70]. This assessment includes identifying significant environmental problems and analyzing the technique in terms of identified ecological problems [71,72]. Life Cycle Assessment is recognized as one of the best tools for this purpose [73,74].

Despite the considerable volume of this article, such a multifaceted topic, which is an ecological and energetic, environment and system control analysis of the life cycle of selected postproduction waste of wind power plant blades, has not yet been fully discussed. Therefore, it seems reasonable to develop the presented considerations in the form of the second part of this article, covering the most important environmental aspects of the grouping phase and weighing (optional steps for assessment) in the framework of the LCA method. The main research task will be to determine the level of negative (or positive) impact of the life cycle of the assessed waste on human health, environmental quality and the depletion of natural resources. The environmental aspects of the assessment will include eleven impact categories specific to the Eco-indicator 99 model. The obtained research results will be additionally grouped and compiled into three areas of influence: human health, ecosystem quality and resources. There will also be four emission areas for specific chemicals including air, water, soil and raw. The CED method will be used for energy analysis. The obtained results will be presented in the LCA unit—Pt (environmental points). One thousand points correspond to the average environmental impact of one European per year.

## Figures and Tables

**Table 1:** Data aggregation method in the method Eco-indicator 99.

**Impact Category**	**Area of Influence**	**ECOLABEL**
Carcinogens	Human health	
Resp. organics		
Resp. inorganics		
Climate change		
Radiation		
Ozone layer		
Ecotoxicity	Ecosystem quality	
Acidification/Eutrophication		
Land use		
Minerals	Resources	
Fossil fuels		

**Table 2:** Characterization results of environmental consequences occurring in the life cycle of selected postproduction waste of wind power plant blades—part 1.

Impact Category	Unit	Fiberglass Mat		Roving Fabric		Resin Discs		Distribution Hoses	
		Life Cycle	Recy-Cling	Life Cycle	Recy-Cling	Life Cycle	Recy-Cling	Life Cycle	Recy-Cling
Carcinogens	DALY per 1 mg	1.15 × 10^−3^	1.27 × 10^−4^	1.54 × 10^−4^	1.21 × 10^−4^	1.00 × 10^−6^	1.27 × 10^−4^	1.20 × 10^−4^	7.46 × 10^−5^
Resp. organics	DALY per 1 mg	5.09 × 10^−6^	−1.77 × 10^−5^	5.95 × 10^−6^	−1.69 × 10^−5^	1.09 × 10^−5^	−1.77 × 10^−5^	2.25 × 10^−5^	−1.54 × 10^−5^
Resp. inorganics	DALY per 1 mg	1.96 × 10^−3^	−3.94 × 10^−4^	3.64 × 10^−3^	−3.74 × 10^−4^	1.70 × 10^−3^	−3.94 × 10^−4^	1.65 × 10^−3^	−1.19 × 10^−3^
Climate change	DALY per 1 mg	1.01 × 10^−3^	−6.95 × 10^−5^	1.74 × 10^−3^	−6.60 × 10^−5^	2.32 × 10^−4^	−6.95 × 10^−5^	5.09 × 10^−4^	−3.14 × 10^−5^
Radiation	DALY per 1 mg	1.91 × 10^−5^	0.00 × 10^0^	1.32 × 10^−5^	0.00 × 10^0^	0.00 × 10^0^	0.00 × 10^0^	1.91 × 10^−6^	0.00 × 10^0^
Ozone layer	DALY per 1 mg	6.04 × 10^−7^	−4.74 × 10^−7^	7.34 × 10^−7^	−4.50 × 10^−7^	0.00 × 10^0^	−4.74 × 10^−7^	6.04 × 10^−8^	−8.62 × 10^−8^
Ecotoxicity	PAF·m^2^/yr. per 1 mg	9.55 × 10^2^	3.32 × 10^2^	3.53 × 10^2^	3.15 × 10^2^	6.96 × 10^2^	3.32 × 10^2^	1.03 × 10^2^	3.30 × 10^2^
Acidification/ Eutrophication	PDF·m^2^/yr. per 1 mg	5.93 × 10^1^	−3.19 × 10^1^	9.84 × 10^1^	−3.03 × 10^1^	9.88 × 10^1^	−3.19 × 10^1^	6.30 × 10^1^	−6.01 × 10^1^
Land use	PDF·m^2^/yr. per 1 mg	5.32 × 10^0^	0.00 × 10^0^	1.66 × 10^1^	0.00 × 10^0^	2.98 × 10^1^	0.00 × 10^0^	3.08 × 10^1^	0.00 × 10^0^
Minerals	MJ surplus per 1 mg	2.02 × 10^1^	−1.40 × 10^−1^	7.07 × 10^0^	−1.33 × 10^−1^	7.80 × 10^−1^	−1.40 × 10^−1^	2.23 × 10^0^	−1.13 × 10^−1^
Fossil fuels	MJ surplus per 1 mg	7.82 × 10^3^	−8.61 × 10^3^	1.40 × 10^4^	−8.18 × 10^3^	3.41 × 10^4^	−8.61 × 10^3^	5.07 × 10^3^	−5.37 × 10^3^

Red highlight—the highest level of negative environmental consequences for a given unit. Green highlight—the highest level of positive environmental consequences for a given unit.

**Table 3:** Characterization results of environmental consequences occurring in the life cycle of selected postproduction waste of wind power plant blades—part 2.

Impact Category	Unit	Spiral Hoses with Resin		Vacuum Bag Film		Infusion Materials Residues		Surplus Materials	
		Life Cycle	Recy-Cling	Life Cycle	Recy-Cling	Life Cycle	Recy-Cling	Life Cycle	Recy-Cling
Carcinogens	DALY per 1 mg	4.65 × 10^−5^	1.10 × 10^−4^	8.75 × 10^−5^	1.27 × 10^−5^	1.31 × 10^−4^	1.13 × 10^−4^	3.13 × 10^−5^	1.25 × 10^−4^
Resp. organics	DALY per 1 mg	1.37 × 10^−5^	−1.70 × 10^−5^	5.17 × 10^−6^	−1.77 × 10^−6^	1.42 × 10^−5^	−1.68 × 10^−5^	1.24 × 10^−5^	−1.44 × 10^−5^
Resp. inorganics	DALY per 1 mg	1.71 × 10^−3^	−6.59 × 10^−4^	1.17 × 10^−3^	−3.94 × 10^−5^	1.86 × 10^−3^	−5.64 × 10^−4^	2.01 × 10^−3^	−4.35 × 10^−4^
Climate change	DALY per 1 mg	3.16 × 10^−4^	−5.68 × 10^−5^	3.82 × 10^−4^	−6.95 × 10^−6^	8.16 × 10^−4^	−5.89 × 10^−5^	4.70 × 10^−4^	−3.88 × 10^−5^
Radiation	DALY per 1 mg	5.47 × 10^−6^	0.00 × 10^0^	3.74 × 10^−5^	0.00 × 10^0^	1.18 × 10^−5^	0.00 × 10^0^	2.10 × 10^−6^	0.00 × 10^0^
Ozone layer	DALY per 1 mg	4.30 × 10^−5^	−3.45 × 10^−7^	1.33 × 10^−6^	−4.74 × 10^−8^	1.85 × 10^−4^	−3.80 × 10^−7^	1.21 × 10^−7^	−5.43 × 10^−7^
Ecotoxicity	PAF·m^2^/yr. per 1 mg	7.43 × 10^1^	3.31 × 10^2^	3.26 × 10^2^	3.32 × 10^1^	1.59 × 10^2^	3.25 × 10^2^	6.17 × 10^1^	3.26 × 10^2^
Acidification/ Eutrophication	PDF·m^2^/yr. per 1 mg	8.75 × 10^1^	−4.13 × 10^1^	3.51 × 10^1^	−3.19 × 10^0^	8.56 × 10^1^	−3.76 × 10^1^	8.69 × 10^1^	−3.11 × 10^1^
Land use	PDF·m^2^/yr. per 1 mg	3.43 × 10^1^	0.00 × 10^0^	5.15 × 10^1^	0.00 × 10^0^	3.78 × 10^1^	0.00 × 10^0^	3.18 × 10^1^	0.00 × 10^0^
Minerals	MJ surplus per 1 mg	5.96 × 10^−1^	−1.31 × 10^−1^	1.07 × 10^1^	−1.40 × 10^−2^	1.38 × 10^0^	−1.32 × 10^−1^	1.73 × 10^0^	−1.57 × 10^−1^
Fossil fuels	MJ surplus per 1 mg	2.55 × 10^4^	−7.53 × 10^3^	2.63 × 10^3^	−8.61 × 10^2^	2.24 × 10^4^	−7.71 × 10^3^	2.12 × 10^4^	−8.26 × 10^3^

Red highlight—the highest level of negative environmental consequences for a given unit. Green highlight—the highest level of positive environmental consequences for a given unit.

**Table 4:** Characterization results of environmental consequences for processes related to greenhouse gas emissions (GHG) for selected postproduction waste of wind power plant blades—part 1 [unit: kg CO_2_ eq per 1 mg].

Substance	Compa-Rtment	Fiberglass Mat		Roving Fabric		Resin Discs		Distribution Hoses	
		Life Cycle	Recy-Cling	Life Cycle	Recy-Cling	Life Cycle	Recy-Cling	Life Cycle	Recy-Cling
Carbon dioxide, in air	Raw	−3.11 × 10^1^	x	−9.11 × 10^1^	x	x	x	−3.11 × 10^0^	x
Carbon dioxide	Air	0.01 × 10^−9^	−3.37 × 10^2^	0.01 × 10^−9^	−3.20 × 10^2^	1.10 × 10^3^	−3.37 × 10^2^	1.80 × 10^3^	−1.26 × 10^2^
Carbon dioxide, biogenic	Air	3.34 × 10^1^	x	6.39 × 10^1^	x	x	x	3.34 × 10^0^	x
Carbon dioxide, fossil	Air	3.19 × 10^3^	x	7.35 × 10^3^	x	x	x	3.19 × 10^2^	x
Carbon monoxide, fossil	Air	6.28 × 10^0^	x	1.05 × 10^1^	x	x	x	x	x
Dinitrogen monoxide	Air	1.22×10^3^	3.19 × 10^0^	2.10 × 10^2^	3.03 × 10^0^	0.01 × 10^−9^	3.19 × 10^0^	1.22 × 10^2^	3.00 × 10^0^
Methane	Air	x	x	x	x	0.01 × 10^−9^	1.91 × 10^0^	1.57 × 10^2^	−2.60 × 10^1^
Methane, dichloro-, HCC-30	Air	x	x	x	x	3.76 × 10^0^	x	x	x
Methane, fossil	Air	2.60 × 10^2^	x	7.68 × 10^2^	x	x	x	2.60 × 10^1^	x
Remaining substances	x	5.40 × 10^0^	1.17 × 10^0^	1.50 × 10^1^	1.12 × 10^0^	5.51 × 10^−1^	−7.31 × 10^−1^	4.42 × 10^0^	−2.93 × 10^0^
**TOTAL**		**4.68** × **10^3^**	−**3.33** × **10^2^**	**8.33** × **10^3^**	−**3.16** × **10^2^**	**1.11** × **10^3^**	−**3.33** × **10^2^**	**2.43** × **10^3^**	−**1.52** × **10^2^**

Red highlight—the highest level of negative environmental consequences for the emissions of the analyzed substances. Green highlight—the highest level of positive environmental consequences for the emissions of the analyzed substances.

**Table 5:** Characterization results of environmental consequences for processes related to greenhouse gas emissions (GHG) for selected postproduction waste of wind power plant blades—part 2 [unit: kg CO_2_ eq per 1 mg].

Substance	Compa-Rtment	Spiral Hoses with Resin		Vacuum Bag Film		Infusion Materials Residues		Surplus Materials	
		Life Cycle	Recy-Cling	Life Cycle	Recy-Cling	Life Cycle	Recy-Cling	Life Cycle	Recy-Cling
Carbon dioxide, in air	Raw	x	x	x	x	x	x	−1.48 × 10^1^	x
Carbon dioxide	Air	1.49 × 10^3^	−2.67 × 10^2^	1.74 × 10^3^	−3.37 × 10^1^	1.50 × 10^3^	−2.80 × 10^2^	9.26 × 10^2^	−1.97 × 10^2^
Carbon dioxide, biogenic	Air	x	x	x	x	x	x	1.05 × 10^1^	x
Carbon dioxide, fossil	Air	x	x	x	x	1.09 × 10^2^	x	1.17 × 10^3^	x
Chloroform	Air	x	x	x	x	8.97 × 10^0^	x	x	x
Dinitrogen monoxide	Air	2.42 × 10^0^	3.13 × 10^0^	1.53 × 10^1^	3.19 × 10^−1^	5.48 × 10^0^	3.09 × 10^0^	3.31 × 10^1^	3.19 × 10^0^
Ethane, 1,2-dichloro-1,1,2,2-tetrafluoro-, CFC-114	Air	x	x	1.97 × 10^0^	x	x	x	x	x
Ethane, 1,1,1,2-tetrafluoro-HFC-134a	Air	x	x	x	x	9.10×10^1^	x	x	x
Methane	Air	5.32 × 10^1^	−7.41 × 10^0^	6.29 × 10^1^	1.91 × 10^−1^	3.67 × 10^1^	−4.18 × 10^0^	x	8.18 × 10^0^
Methane, chlorodifluoro-, HCFC-22	Air	x	x	x	x	1.69 × 10^3^	x	x	x
Methane, dichloro-, HCC-30	Air	2.63 × 10^0^	x	x	x	x	x	x	x
Methane, dichlorodifluoro-, CFC-12	Air	x	x	x	x	2.44 × 10^2^	x	x	x
Methane, fossil	Air	x	x	x	x	6.39 × 10^0^	x	1.22 × 10^2^	x
Methane, tetrachloro-, CFC-10	Air	6.07 × 10^1^	x	x	x	1.74 × 10^2^	x	x	x
Methane, trifluoro-, HFC-23	Air	x	x	x	x	7.60 × 10^2^	x	x	x
Remaining substances	x	2.11 × 10^0^	−1.47 × 10^0^	1.84 × 10^0^	−7.31 × 10^−2^	6.51 × 10^0^	−1.21 × 10^0^	6.49 × 10^0^	−7.10 × 10^−1^
**TOTAL**		**1.61** × **10^3^**	−**2.73** × **10^2^**	**1.83** × **10^3^**	−**3.33** × **10^1^**	**4.63** × **10^3^**	−**2.82** × **10^2^**	**2.25** × **10^3^**	−**1.86** × **10^2^**

Red highlight—the highest level of negative environmental consequences for the emissions of the analyzed substances. Green highlight—the highest level of positive environmental consequences for the emissions of the analyzed substances.

**Table 6:** Characterization results of environmental consequences for processes related to energy production for selected postproduction waste of wind power plant blades—part 1 [unit: MJ eq per 1 mg].

Impact Category	Fiberglass Mat		Roving Fabric		Resin Discs		Distribution Hoses	
	Life Cycle	Recy-Cling	Life Cycle	Recy-Cling	Life Cycle	Recy-Cling	Life Cycle	Recy-Cling
Non renewable, fossil	1.13 × 10^4^	−8.31 × 10^3^	2.03 × 10^4^	−7.89 × 10^3^	3.93 × 10^4^	−8.31 × 10^3^	8.37 × 10^3^	−5.14 × 10^3^
Non-renewable, nuclear	1.69 × 10^3^	3.15 × 10^3^	2.94 × 10^3^	2.99 × 10^3^	0.00 × 10^0^	3.15 × 10^3^	1.03 × 10^3^	2.32 × 10^3^
Renewable, biomass	5.72 × 10^1^	0.00 × 10^0^	3.12 × 10^2^	0.00 × 10^0^	0.00 × 10^0^	0.00 × 10^0^	5.72 × 10^0^	0.00 × 10^0^
Renewable, wind, solar, geothermal	2.86 × 10^1^	0.00 × 10^0^	2.01 × 10^1^	0.00 × 10^0^	0.00 × 10^0^	0.00 × 10^0^	2.86 × 10^0^	0.00 × 10^0^
Renewable, water	1.92 × 10^2^	4.22 × 10^2^	2.19 × 10^2^	4.01 × 10^2^	0.00 × 10^0^	4.22 × 10^2^	1.53 × 10^2^	3.71 × 10^2^
**TOTAL**	**1.33** × **10^4^**	−**4.74** × **10^3^**	**2.38** × **10^4^**	−**4.50** × **10^3^**	**3.93** × **10^4^**	−**4.74** × **10^3^**	**9.57** × **10^3^**	−**2.45** × **10^3^**

Red highlight—the highest level of negative environmental consequences for the analyzed impact categories. Green highlight—the highest level of positive environmental consequences for the analyzed impact categories.

**Table 7:** Characterization results of environmental consequences for processes related to energy production for selected postproduction waste of wind power plant blades—part 2 [unit: MJ eq per 1 mg].

Impact Category	Spiral Hoses with Resin		Vacuum Bag Film		Infusion Materials Residues		Surplus Materials	
	Life Cycle	Recy-Cling	Life Cycle	Recy-Cling	Life Cycle	Recy-Cling	Life Cycle	Recy-Cling
Non renewable, fossil	3.02 × 10^4^	−7.25 × 10^3^	4.74 × 10^3^	−8.31 × 10^2^	2.71 × 10^4^	−7.43 × 10^3^	2.60 × 10^4^	−7.92 × 10^3^
Non-renewable, nuclear	5.20 × 10^2^	2.87 × 10^3^	2.24 × 10^3^	3.15 × 10^2^	7.31 × 10^2^	2.90 × 10^3^	5.29 × 10^2^	3.15 × 10^3^
Renewable, biomass	0.00 × 10^0^	0.00 × 10^0^	0.00 × 10^0^	0.00 × 10^0^	6.22 × 10^0^	0.00 × 10^0^	5.00 × 10^1^	0.00 × 10^0^
Renewable, wind, solar, geothermal	0.00 × 10^0^	0.00 × 10^0^	0.00 × 10^0^	0.00 × 10^0^	1.45 × 10^0^	0.00 × 10^0^	3.20 × 10^0^	0.00 × 10^0^
Renewable, water	6.76 × 10^1^	4.05 × 10^2^	2.61 × 10^2^	4.22 × 10^1^	9.15 × 10^1^	4.02 × 10^2^	3.63 × 10^1^	3.98 × 10^2^
**TOTAL**	**3.08** × **10^4^**	−**3.97** × **10^3^**	**7.24** × **10^3^**	−**4.74** × **10^2^**	**2.79** × **10^4^**	−**4.12** × **10^3^**	**2.66** × **10^4^**	−**4.37** × **10^3^**

Red highlight—the highest level of negative environmental consequences for the analyzed impact categories. Green highlight—the highest level of positive environmental consequences for the analyzed impact categories.

## Supplementary Materials

The following are available online at https://www.mdpi.com/2073-4360/12/8/1828/s1. Table S1: Characterization results of environmental consequences for carcinogens present in selected postproduction waste of wind power plant blades—part 1 [unit: DALY per 1 mg]. Table S2: Characterization results of environmental consequences for carcinogens present in selected postproduction waste of wind power plant blades—part 2 [unit: DALY per 1 mg]. Table S3: Characterization results of environmental consequences for organic compounds causing respiratory diseases present in selected postproduction waste of wind power plant blades—part 1 [unit: DALY per 1 mg]. Table S4: Characterization results of environmental consequences for organic compounds causing respiratory diseases present in selected postproduction waste of wind power plant blades—part 2 [unit: DALY per 1 mg]. Table S5: Characterization results of environmental consequences for inorganic compounds causing respiratory diseases present in selected postproduction waste of wind farm blades—part 1 [unit: DALY per 1 mg]. Table S6: Characterization results of environmental consequences for inorganic compounds causing respiratory diseases present in selected postproduction waste of wind farm blades—part 2 [unit: DALY per 1 mg]. Table S7: Characterization results of environmental consequences for compounds causing climate change present in selected postproduction waste of wind power plant blades—part 1 [unit: DALY per 1 mg]. Table S8: Characterization results of environmental consequences for compounds causing climate change present in selected postproduction waste of wind power plant blades—part 2 [unit: DALY per 1 mg]. Table S9: Characterization results of environmental consequences for radioactive substances present in selected postproduction waste of wind power plant blades—part 1 [unit: DALY per 1 mg]. Table S10: Characterization results of environmental consequences for radioactive substances present in selected postproduction waste of wind power plant blades—part 2 [unit: DALY per 1 mg]. Table S11: Characterization results of environmental consequences for compounds causing an increase in the ozone hole, present in selected postproduction waste of wind power plant blades—part 1 [unit: DALY per 1 mg]. Table S12: Characterization results of environmental consequences for compounds causing an increase in the ozone hole present in selected postproduction waste of wind power plant blades—part 2 [unit: DALY per 1 mg]. Table S13: Characterization results of environmental consequences for eco toxic compounds present in selected postproduction waste of wind power plant blades—part 1 [unit: PAF·m^2^/yr. per 1 mg]. Table S14: Characterization results of environmental consequences for eco toxic compounds present in selected postproduction waste of wind power plant blades—part 2 [unit: PAF·m^2^/yr. per 1 mg]. Table S15: Characterization results of environmental consequences for compounds causing acidification or eutrophication present in selected postproduction waste of wind power plant blades—part 1 [unit: PDF·m^2^/yr. per 1 mg]. Table S16: Characterization results of environmental consequences for compounds causing acidification or eutrophication present in selected postproduction waste of wind power plant blades—part 2 [unit: PDF·m^2^/yr. per 1 mg]. Table S17: Characterization results of environmental consequences for processes related to land use present in selected postproduction waste of wind power plants blades—part 1 [unit: PDF·m^2^/yr. per 1 mg]. Table S18: Characterization results of environmental consequences for processes related to land use present in selected postproduction waste of wind power plant blades—part 2 [unit: PDF·m^2^/yr per 1 mg]. Table S19: Characterization results of environmental consequences for processes related to the extraction of mineral resources present in selected postproduction waste of wind power plant blades—part 1 [unit: MJ surplus per 1 mg]. Table S20: Characterization results of environmental consequences for processes related to the extraction of mineral resources present in selected postproduction waste of wind power plant blades—part 2 [unit: MJ surplus per 1 mg]. Table S21: Characterization results of environmental consequences for processes related to the extraction of fossil fuels present in selected postproduction waste of wind power plant blades—part 1 [unit: MJ surplus per 1 mg]. Table S22: Characterization results of environmental consequences for processes related to the extraction of fossil fuels present in selected postproduction waste of wind power plant blades—part 2 [unit: MJ surplus per 1 mg].
