# Supplementary material for: Control the System and Environment of Post-Production Wind Turbine Blade Waste Using Life Cycle Models. Part 1. Environmental Transformation Models
Source: Polymers (Basel). 2020 Aug 14;12(8):1828. doi: 10.3390/polym12081828 (PMC7464857; doi:10.3390/polym12081828)
Supplement: Supplementary file 1 [file polymers-12-01828-s001.pdf]

polymers

MDPI

Supplementary

Control the System and Environment of Post-Production Wind Turbine Blade Waste Using Life Cycle Models. Part 1. Environmental Transformation Models

Izabela Piasecka [1,*], Patrycja Bałdowska-Witos [1,*], Józef Flizikowski [1], Katarzyna Piotrowska [2] and Andrzej Tomporowski [1]

[1] Faculty of Mechanical Engineering, University of Science and Technology in Bydgoszcz, 85-796 Bydgoszcz, Poland; izabela.piasecka@utp.edu.pl (I.P); patrycja.baldowska-witos@utp.edu.pl (P.B.-W.); a.tomporowski@utp.edu.pl (A.T.)
[2] Faculty of Mechanical Engineering, Lublin University of Technology, 20-618 Lublin, Poland; k.piotrowska@pollub.pl (K.P)
* Correspondence: izabela.piasecka@utp.edu.pl (I.P); patrycja.baldowska-witos@utp.edu.pl (P.B.-W.)

Received: date; Accepted: date; Published: date

For all tables:
red highlight – the highest level of negative environmental consequences for a given unit,
green highlight – the highest level of positive environmental consequences for a given unit.

Table S1. Characterization results of environmental consequences for carcinogens present in selected post-production waste of wind power plant blades – part 1 [unit: DALY per 1 Mg].

SUBSTANCE	COMPARTMENT	FIBERGLASS MAT		ROVING FABRIC		RESIN DISCS		DISTRIBUTION HOSES	
		LIFE CYCLE	RECYCLING	LIFE CYCLE	RECYCLING	LIFE CYCLE	RECYCLING	LIFE CYCLE	RECYCLING
Arsenic	Air	$3.29 \cdot 10^{-4}$	x	$1.15 \cdot 10^{-6}$	x	x	x	$3.29 \cdot 10^{-5}$	x
Arsenic	Soil	$1.19 \cdot 10^{-5}$	x	x	x	x	x	$1.19 \cdot 10^{-6}$	x
Arsenic, ion	Water	$1.40 \cdot 10^{-4}$	$7.34 \cdot 10^{-5}$	$1.11 \cdot 10^{-4}$	$6.97 \cdot 10^{-5}$	$0.01 \cdot 10^{-9}$	$7.34 \cdot 10^{-5}$	$1.40 \cdot 10^{-5}$	$1.91 \cdot 10^{-5}$
Cadmium	Air	$6.03 \cdot 10^{-4}$	$2.64 \cdot 10^{-6}$	$2.88 \cdot 10^{-6}$	$2.51 \cdot 10^{-6}$	$0.01 \cdot 10^{-9}$	$2.64 \cdot 10^{-6}$	$6.03 \cdot 10^{-5}$	$2.86 \cdot 10^{-6}$
Cadmium	Soil	$4.04 \cdot 10^{-5}$	x	$8.65 \cdot 10^{-7}$	x	x	x	$4.04 \cdot 10^{-6}$	x
Cadmium, ion	Water	$1.12 \cdot 10^{-5}$	$1.60 \cdot 10^{-6}$	$2.54 \cdot 10^{-5}$	$1.52 \cdot 10^{-6}$	$0.01 \cdot 10^{-9}$	$1.60 \cdot 10^{-6}$	$1.12 \cdot 10^{-6}$	$6.19 \cdot 10^{-7}$
Ethane, 1,2-dichloro-	Air	x	x	$4.75 \cdot 10^{-7}$	x	x	x	x	x
Metallic ions, unspecified	Water	$0.01 \cdot 10^{-9}$	$-6.82 \cdot 10^{-6}$	$0.01 \cdot 10^{-9}$	$-6.48 \cdot 10^{-6}$	$8.33 \cdot 10^{-7}$	$-6.82 \cdot 10^{-6}$	$2.65 \cdot 10^{-6}$	$-3.36 \cdot 10^{-6}$
Metals, unspecified	Air	$0.01 \cdot 10^{-9}$	$5.65 \cdot 10^{-5}$	$0.01 \cdot 10^{-9}$	$5.37 \cdot 10^{-5}$	$0.01 \cdot 10^{-9}$	$5.65 \cdot 10^{-5}$	$1.88 \cdot 10^{-6}$	$5.54 \cdot 10^{-5}$
Methane, dichloro-, HCC-30	Air	x	x	x	x	$1.64 \cdot 10^{-7}$	x	x	x
PAH, polycyclic aromatic hydrocarbons	Water	x	x	x	x	$0.01 \cdot 10^{-9}$	$-2.79 \cdot 10^{-7}$	x	x
Particulates, < 2.5 µm	Air	$5.29 \cdot 10^{-6}$	x	$1.07 \cdot 10^{-5}$	x	x	x	$5.29 \cdot 10^{-7}$	x
Propylene oxide	Air	$2.40 \cdot 10^{-6}$	x	x	x	x	x	$2.40 \cdot 10^{-7}$	x
Propylene oxide	Water	$8.57 \cdot 10^{-6}$	x	x	x	x	x	$8.57 \cdot 10^{-7}$	x

Remaining substances	x	$2.15\cdot10^{-6}$	$-2.14\cdot10^{-7}$	$6.53\cdot10^{-7}$	$-2.04\cdot10^{-7}$	$4.16\cdot10^{-9}$	$6.50\cdot10^{-8}$	$2.15\cdot10^{-7}$	$2.61\cdot10^{-8}$
**TOTAL**		**$1.15\cdot10^{-3}$**	**$1.27\cdot10^{-4}$**	**$1.54\cdot10^{-4}$**	**$1.21\cdot10^{-4}$**	**$1.00\cdot10^{-6}$**	**$1.27\cdot10^{-4}$**	**$1.20\cdot10^{-4}$**	**$7.46\cdot10^{-5}$**

Table S2. Characterization results of environmental consequences for carcinogens present in selected post-production waste of wind power plant blades – part 2 [unit: DALY per 1 Mg].

SUBSTANCE	COMPARTMENT	SPIRAL HOSES WITH RESIN		VACUUM BAG FILM		INFUSION MATERIALS RESIDUES		SURPLUS MATERIALS	
		LIFE CYCLE	RECYCLING	LIFE CYCLE	RECYCLING	LIFE CYCLE	RECYCLING	LIFE CYCLE	RECYCLING
Arsenic	Air	$5.36\cdot10^{-7}$	x	$2.87\cdot10^{-6}$	x	$1.21\cdot10^{-6}$	x	$1.94\cdot10^{-7}$	x
Arsenic, ion	Water	$9.01\cdot10^{-6}$	$5.53\cdot10^{-5}$	$6.05\cdot10^{-5}$	$7.34\cdot10^{-6}$	$2.15\cdot10^{-5}$	$5.99\cdot10^{-5}$	$1.77\cdot10^{-5}$	$7.31\cdot10^{-5}$
Cadmium	Air	$4.47\cdot10^{-6}$	$2.72\cdot10^{-6}$	$1.91\cdot10^{-5}$	$2.64\cdot10^{-7}$	$9.54\cdot10^{-6}$	$2.64\cdot10^{-6}$	$4.82\cdot10^{-7}$	$2.53\cdot10^{-6}$
Cadmium, ion	Water	$6.97\cdot10^{-7}$	$1.27\cdot10^{-6}$	$2.90\cdot10^{-6}$	$1.60\cdot10^{-7}$	$2.51\cdot10^{-6}$	$1.35\cdot10^{-6}$	$4.18\cdot10^{-6}$	$1.51\cdot10^{-6}$
Chloroform	Air	x	x	x	x	$7.86\cdot10^{-6}$	x	x	x
Chloroform	Water	$1.17\cdot10^{-6}$	x	x	x	$2.34\cdot10^{-6}$	x	x	x
Metallic ions, unspecified	Water	$1.17\cdot10^{-6}$	$-5.67\cdot10^{-6}$	$1.07\cdot10^{-6}$	$-6.82\cdot10^{-7}$	$2.83\cdot10^{-6}$	$-5.92\cdot10^{-6}$	$5.35\cdot10^{-6}$	$-6.75\cdot10^{-6}$
Metals, unspecified	Air	$4.18\cdot10^{-7}$	$5.61\cdot10^{-5}$	$3.48\cdot10^{-7}$	$5.65\cdot10^{-6}$	$7.67\cdot10^{-7}$	$5.51\cdot10^{-5}$	$1.36\cdot10^{-6}$	$5.50\cdot10^{-5}$
Methane, tetrachloro-, CFC-10	Air	$2.82\cdot10^{-5}$	x	x	x	$8.08\cdot10^{-5}$	x	x	x
Methane, tetrachloro-, CFC-10	Water	$2.45\cdot10^{-7}$	x	x	x	$4.90\cdot10^{-7}$	x	x	x
Nickel	Air	x	x	$1.46\cdot10^{-7}$	$6.03\cdot10^{-9}$	x	x	x	x
PAH, polycyclic aromatic hydrocarbons	Water	x	x	$4.01\cdot10^{-7}$	$-2.79\cdot10^{-8}$	x	x	$8.30\cdot10^{-9}$	$-3.12\cdot10^{-7}$
Particulates, < 2.5 μm	Air	x	x	x	x	x	x	$1.70\cdot10^{-6}$	x
Remaining substances	x	$5.56\cdot10^{-7}$	$-1.34\cdot10^{-7}$	$1.20\cdot10^{-7}$	$4.71\cdot10^{-10}$	$1.07\cdot10^{-6}$	$-1.57\cdot10^{-7}$	$3.85\cdot10^{-7}$	$6.45\cdot10^{-8}$
**TOTAL**		**$4.65\cdot10^{-5}$**	**$1.10\cdot10^{-4}$**	**$8.75\cdot10^{-5}$**	**$1.27\cdot10^{-5}$**	**$1.31\cdot10^{-4}$**	**$1.13\cdot10^{-4}$**	**$3.13\cdot10^{-5}$**	**$1.25\cdot10^{-4}$**

Table S3. Characterization results of environmental consequences for organic compounds causing respiratory diseases present in selected post-production waste of wind power plant blades – part 1 [unit: DALY per 1 Mg].

SUBSTANCE	COMPARTMENT	FIBERGLASS MAT		ROVING FABRIC		RESIN DISCS		DISTRIBUTION HOSES	
		LIFE CYCLE	RECYCLING	LIFE CYCLE	RECYCLING	LIFE CYCLE	RECYCLING	LIFE CYCLE	RECYCLING
Acetaldehyde	Air	$2.59\cdot10^{-8}$	x	x	x	x	x	x	x
Acetic acid	Air	$6.52\cdot10^{-8}$	x	x	x	x	x	x	x
Benzene	Air	$1.12\cdot10^{-7}$	$-2.25\cdot10^{-10}$	$1.96\cdot10^{-8}$	$-2.14\cdot10^{-10}$	x	x	$1.12\cdot10^{-8}$	$5.33\cdot10^{-10}$
Butane	Air	$1.93\cdot10^{-8}$	x	x	x	x	x	x	x
Butene	Air	x	x	$1.17\cdot10^{-8}$	x	x	x	x	x
Cumene	Air	$6.96\cdot10^{-8}$	x	$1.20\cdot10^{-8}$	x	x	x	x	x
Ethane	Air	$2.79\cdot10^{-8}$	x	$1.32\cdot10^{-8}$	x	x	x	x	x
Ethene	Air	$4.84\cdot10^{-8}$	x	$2.22\cdot10^{-8}$	x	x	x	x	x
Hydrocarbons, aliphatic,	Air	$8.22\cdot10^{-8}$	x	$2.58\cdot10^{-8}$	x	x	x	$8.22\cdot10^{-9}$	x

alkanes, unspecified									
Hydrocarbons, aromatic	Air	$1.69\cdot10^{-7}$	$1.70\cdot10^{-8}$	$5.30\cdot10^{-8}$	$1.62\cdot10^{-8}$	x	$1.70\cdot10^{-8}$	$1.69\cdot10^{-8}$	$1.99\cdot10^{-8}$
Hydrocarbons, chlorinated	Air	x	x	x	x	$1.31\cdot10^{-7}$	$4.10\cdot10^{-13}$	$4.40\cdot10^{-12}$	$-2.04\cdot10^{-7}$
Hydrocarbons, unspecified	Air	$0.01\cdot10^{-9}$	$1.28\cdot10^{-7}$	$0.01\cdot10^{-9}$	$1.22\cdot10^{-7}$	$1.07\cdot10^{-5}$	$1.28\cdot10^{-7}$	$2.19\cdot10^{-5}$	$1.28\cdot10^{-7}$
Methane	Air	x	x	x	x	x	x	$8.76\cdot10^{-8}$	$-1.45\cdot10^{-8}$
Methane, dichloro-, HCC-30	Air	x	x	x	x	$5.44\cdot10^{-8}$	x	x	x
Methane, fossil	Air	$1.45\cdot10^{-7}$	x	$4.27\cdot10^{-7}$	x	x	x	$1.45\cdot10^{-8}$	x
Methane, tetrachloro-, CFC-10	Air	x	x	x	x	x	x	x	x
Methanol	Air	$3.51\cdot10^{-8}$	x	x	x	x	x	x	x
NMVOC, unspecified origin	Air	$3.67\cdot10^{-6}$	$-1.79\cdot10^{-5}$	$5.24\cdot10^{-6}$	$-1.70\cdot10^{-5}$	$0.02\cdot10^{-9}$	$-1.79\cdot10^{-5}$	$3.67\cdot10^{-7}$	$-1.53\cdot10^{-5}$
Pentane	Air	$2.68\cdot10^{-8}$	x	$1.16\cdot10^{-8}$	x	x	x	x	x
Phenol	Air	$9.26\cdot10^{-8}$	x	x	x	x	x	$9.26\cdot10^{-9}$	x
Propane	Air	$1.60\cdot10^{-8}$	x	x	x	x	x	x	x
Propene	Air	$4.35\cdot10^{-7}$	x	$3.04\cdot10^{-8}$	x	x	x	$4.35\cdot10^{-8}$	x
Xylene	Air	$1.77\cdot10^{-8}$	x	$1.22\cdot10^{-8}$	x	x	x	x	x
Remaining substances	x	$3.64\cdot10^{-8}$	$-5.18\cdot10^{-9}$	$7.89\cdot10^{-8}$	$-4.92\cdot10^{-9}$	$2.89\cdot10^{-10}$	$-5.40\cdot10^{-9}$	$3.88\cdot10^{-8}$	$-5.66\cdot10^{-10}$
**TOTAL**		**$5.09\cdot10^{-6}$**	**$-1.77\cdot10^{-5}$**	**$5.95\cdot10^{-6}$**	**$-1.69\cdot10^{-5}$**	**$1.09\cdot10^{-5}$**	**$-1.77\cdot10^{-5}$**	**$2.25\cdot10^{-5}$**	**$-1.54\cdot10^{-5}$**

Table S4. Characterization results of environmental consequences for organic compounds causing respiratory diseases present in selected post-production waste of wind power plant blades – part 2 [unit: DALY per 1 Mg].

SUBSTANCE	COMPA-RTMENT	SPIRAL HOSES WITH RESIN		VACUUM BAG FILM		INFUSION MATERIALS RESIDUES		SURPLUS MATERIALS	
		LIFE CYCLE	RECY-CLING	LIFE CYCLE	RECY-CLING	LIFE CYCLE	RECY-CLING	LIFE CYCLE	RECY-CLING
Aldehydes, unspecified	Air	$2.06\cdot10^{-10}$	$-4.41\cdot10^{-9}$	x	x	$1.71\cdot10^{-10}$	$-4.85\cdot10^{-9}$	$8.21\cdot10^{-10}$	$-3.84\cdot10^{-9}$
Benzene	Air	$1.39\cdot10^{-8}$	$2.77\cdot10^{-11}$	x	x	$2.81\cdot10^{-8}$	$-5.37\cdot10^{-11}$	$3.11\cdot10^{-9}$	$-2.87\cdot10^{-10}$
Butane	Air	$7.12\cdot10^{-9}$	x	$1.52\cdot10^{-8}$	x	$1.52\cdot10^{-8}$	x	x	x
Chloroform	Air	x	x	x	x	$1.48\cdot10^{-8}$	x	x	x
Ethane	Air	x	x	x	x	$3.41\cdot10^{-9}$	x	$2.19\cdot10^{-9}$	x
Ethene	Air	$2.14\cdot10^{-8}$	x	$4.02\cdot10^{-9}$	x	$4.38\cdot10^{-8}$	x	$3.70\cdot10^{-9}$	x
Formaldehyde	Air	x	x	$4.08\cdot10^{-9}$	x	x	x	x	x
Heptane	Air	x	x	$4.64\cdot10^{-9}$	x	$4.36\cdot10^{-9}$	x	x	x
Hexane	Air	$4.11\cdot10^{-9}$	x	$8.95\cdot10^{-9}$	x	$8.59\cdot10^{-9}$	x	x	x
Hydrocarbons, aliphatic, alkanes, unspecified	Air	x	x	$6.12\cdot10^{-9}$	x	$7.11\cdot10^{-9}$	x	$4.66\cdot10^{-9}$	x
Hydrocarbons, aliphatic, alkenes, unspecified	Air	x	x	$3.42\cdot10^{-9}$	x	x	x	x	x
Hydrocarbons, aromatic	Air	$7.57\cdot10^{-11}$	$1.80\cdot10^{-8}$	x	x	$6.93\cdot10^{-10}$	$1.73\cdot10^{-8}$	$8.41\cdot10^{-9}$	$1.62\cdot10^{-8}$

Hydrocarbons, chlorinated	Air	$9.17\cdot10^{-8}$	$-6.80\cdot10^{-8}$	x	x	$7.26\cdot10^{-8}$	$-4.54\cdot10^{-8}$	$6.04\cdot10^{-8}$	$4.06\cdot10^{-13}$
Hydrocarbons, unspecified	Air	$1.24\cdot10^{-5}$	$1.28\cdot10^{-7}$	$2.69\cdot10^{-6}$	$1.28\cdot10^{-8}$	$1.17\cdot10^{-5}$	$1.25\cdot10^{-7}$	$1.14\cdot10^{-5}$	$1.27\cdot10^{-7}$
Methane	Air	$2.96\cdot10^{-8}$	$-4.12\cdot10^{-9}$	$3.50\cdot10^{-8}$	$1.06\cdot10^{-10}$	$2.04\cdot10^{-8}$	$-2.32\cdot10^{-9}$	x	$4.55\cdot10^{-9}$
Methane, dichloro-, HCC-30	Air	$3.81\cdot10^{-8}$	x	x	x	$3.13\cdot10^{-8}$	x	$2.45\cdot10^{-8}$	x
Methane, fossil	Air	x	x	x	x	$3.56\cdot10^{-9}$	x	$6.77\cdot10^{-8}$	x
Methane, tetrachloro-, CFC-10	Air	$1.18\cdot10^{-8}$	x	x	x	$3.37\cdot10^{-8}$	x	x	x
Methanol	Air	x	x	x	x	$2.55\cdot10^{-8}$	x	x	x
NMVOC, unspecified origin	Air	$1.04\cdot10^{-6}$	$-1.70\cdot10^{-5}$	$2.33\cdot10^{-6}$	$-1.79\cdot10^{-6}$	$2.14\cdot10^{-6}$	$-1.69\cdot10^{-5}$	$8.30\cdot10^{-7}$	$-1.46\cdot10^{-5}$
Pentane	Air	$9.13\cdot10^{-9}$	x	$2.20\cdot10^{-8}$	x	$1.95\cdot10^{-8}$	x	x	x
Phenol	Air	x	x	x	x	x	x	x	x
Propane	Air	x	x	$7.89\cdot10^{-9}$	x	$7.30\cdot10^{-9}$	x	x	x
Propene	Air	$8.97\cdot10^{-9}$	x	x	x	$1.81\cdot10^{-8}$	x	$4.82\cdot10^{-9}$	x
Toluene	Air	x	x	$5.02\cdot10^{-9}$	x	$3.97\cdot10^{-9}$	x	x	x
Xylene	Air	$3.76\cdot10^{-9}$	x	$1.80\cdot10^{-8}$	x	$8.36\cdot10^{-9}$	x	x	x
Remaining substances	x	$1.30\cdot10^{-8}$	$6.10\cdot10^{-11}$	$1.41\cdot10^{-8}$	$1.06\cdot10^{-9}$	$6.44\cdot10^{-9}$	$5.93\cdot10^{-11}$	$1.83\cdot10^{-8}$	$5.64\cdot10^{-11}$
**TOTAL**		**$1.37\cdot10^{-5}$**	**$-1.70\cdot10^{-5}$**	**$5.17\cdot10^{-6}$**	**$-1.77\cdot10^{-6}$**	**$1.42\cdot10^{-5}$**	**$-1.68\cdot10^{-5}$**	**$1.24\cdot10^{-5}$**	**$-1.44\cdot10^{-5}$**

Table S5. Characterization results of environmental consequences for inorganic compounds causing respiratory diseases present in selected post-production waste of wind farm blades – part 1 [unit: DALY per 1 Mg].

SUBSTANCE	COMPA-RTMENT	FIBERGLASS MAT		ROVING FABRIC		RESIN DISCS		DISTRIBUTION HOSES	
		LIFE CYCLE	RECY-CLING	LIFE CYCLE	RECY-CLING	LIFE CYCLE	RECY-CLING	LIFE CYCLE	RECY-CLING
Ammonia	Air	$8.42\cdot10^{-6}$	$3.12\cdot10^{-7}$	$2.97\cdot10^{-6}$	$2.96\cdot10^{-7}$	x	x	$8.42\cdot10^{-7}$	$2.78\cdot10^{-7}$
Nitric oxide	Air	x	x	x	x	$2.21\cdot10^{-6}$	x	x	x
Nitrogen oxides	Air	$7.10\cdot10^{-4}$	$-5.33\cdot10^{-4}$	$1.20\cdot10^{-3}$	$-5.07\cdot10^{-4}$	$1.51\cdot10^{-3}$	$-5.33\cdot10^{-4}$	$8.37\cdot10^{-4}$	$-8.93\cdot10^{-4}$
Particulates	Air	$0.01\cdot10^{-9}$	$1.53\cdot10^{-5}$	$0.01\cdot10^{-9}$	$1.46\cdot10^{-5}$	$0.01\cdot10^{-9}$	$1.53\cdot10^{-5}$	$2.87\cdot10^{-4}$	$-1.54\cdot10^{-4}$
Particulates, < 2.5 µm	Air	$3.79\cdot10^{-4}$	x	$7.65\cdot10^{-4}$	x	x	x	$3.79\cdot10^{-5}$	x
Particulates, > 2.5 um, and < 10 µm	Air	$2.36\cdot10^{-4}$	x	$5.89\cdot10^{-4}$	x	x	x	$2.36\cdot10^{-5}$	x
Particulates, SPM	Air	x	x	x	x	$1.07\cdot10^{-4}$	x	x	x
Sulfate	Air	$4.53\cdot10^{-5}$	x	x	x	x	x	$4.53\cdot10^{-6}$	x
Sulfur dioxide	Air	$5.87\cdot10^{-4}$	x	$1.08\cdot10^{-3}$	x	$7.49\cdot10^{-5}$	x	$5.87\cdot10^{-5}$	x
Sulfur oxides	Air	$0.01\cdot10^{-9}$	$1.24\cdot10^{-4}$	$0.01\cdot10^{-9}$	$1.17\cdot10^{-4}$	$1.28\cdot10^{-5}$	$1.24\cdot10^{-4}$	$4.03\cdot10^{-4}$	$-1.42\cdot10^{-4}$
Remaining substances	x	$0.00\cdot10^{0}$	$1.08\cdot10^{-19}$	$1.65\cdot10^{-6}$	$0.00\cdot10^{0}$	$3.88\cdot10^{-8}$	$3.12\cdot10^{-7}$	$0.00\cdot10^{0}$	$0.00\cdot10^{0}$
**TOTAL**		**$1.96\cdot10^{-3}$**	**$-3.94\cdot10^{-4}$**	**$3.64\cdot10^{-3}$**	**$-3.74\cdot10^{-4}$**	**$1.70\cdot10^{-3}$**	**$-3.94\cdot10^{-4}$**	**$1.65\cdot10^{-3}$**	**$-1.19\cdot10^{-3}$**

Table S6. Characterization results of environmental consequences for inorganic compounds causing respiratory diseases present in selected post-production waste of wind farm blades – part 2 [unit: DALY per 1 Mg].

SUBSTANCE	COMPARTMENT	SPIRAL HOSES WITH RESIN		VACUUM BAG FILM		INFUSION MATERIALS RESIDUES		SURPLUS MATERIALS	
		LIFE CYCLE	RECYCLING	LIFE CYCLE	RECYCLING	LIFE CYCLE	RECYCLING	LIFE CYCLE	RECYCLING
Ammonia	Air	x	x	x	x	x	x	x	x
Nitric oxide	Air	$1.55\cdot10^{-6}$	x	x	x	x	x	x	x
Nitrogen oxides	Air	$1.28\cdot10^{-3}$	$-6.53\cdot10^{-4}$	$3.71\cdot10^{-4}$	$-5.33\cdot10^{-5}$	$1.20\cdot10^{-3}$	$-6.02\cdot10^{-4}$	$1.22\cdot10^{-3}$	$-4.98\cdot10^{-4}$
Particulates	Air	$6.38\cdot10^{-5}$	$-4.11\cdot10^{-5}$	x	$1.53\cdot10^{-6}$	$0.01\cdot10^{-9}$	$-2.26\cdot10^{-5}$	$0.01\cdot10^{-9}$	$1.52\cdot10^{-5}$
Particulates, < 2.5 µm	Air	x	x	x	x	$1.28\cdot10^{-5}$	x	$1.21\cdot10^{-4}$	x
Particulates, > 2.5 µm, and < 10 µm	Air	x	x	x	x	$5.02\cdot10^{-6}$	x	$9.34\cdot10^{-5}$	x
Particulates, < 10 µm (mobile)	Air	$1.09\cdot10^{-6}$	x	$3.68\cdot10^{-6}$	x	$2.20\cdot10^{-6}$	x	x	x
Particulates, < 10 µm (stationary)	Air	$3.67\cdot10^{-5}$	x	$1.80\cdot10^{-4}$	x	$7.46\cdot10^{-5}$	x	x	x
Particulates, SPM	Air	$7.51\cdot10^{-5}$	x	$3.30\cdot10^{-5}$	x	$1.31\cdot10^{-4}$	x	$1.35\cdot10^{-4}$	x
Sulfate	Air	x	x	x	x	$1.36\cdot10^{-5}$	x	x	x
Sulfur dioxide	Air	$5.24\cdot10^{-5}$	x	x	x	$9.49\cdot10^{-5}$	x	$2.05\cdot10^{-4}$	x
Sulfur oxides	Air	$2.02\cdot10^{-4}$	$3.52\cdot10^{-5}$	$5.86\cdot10^{-4}$	$1.24\cdot10^{-5}$	$3.27\cdot10^{-4}$	$6.02\cdot10^{-5}$	$2.39\cdot10^{-4}$	$4.77\cdot10^{-5}$
Remaining substances	x	$7.65\cdot10^{-8}$	$3.01\cdot10^{-7}$	$3.42\cdot10^{-7}$	$3.12\cdot10^{-8}$	$1.45\cdot10^{-6}$	$2.98\cdot10^{-7}$	$1.75\cdot10^{-6}$	$3.14\cdot10^{-7}$

Table S7. Characterization results of environmental consequences for compounds causing climate change, present in selected post-production waste of wind power plant blades – part 1 [unit: DALY per 1 Mg].

SUBSTANCE	COMPARTMENT	FIBERGLASS MAT		ROVING FABRIC		RESIN DISCS		DISTRIBUTION HOSES	
		LIFE CYCLE	RECYCLING	LIFE CYCLE	RECYCLING	LIFE CYCLE	RECYCLING	LIFE CYCLE	RECYCLING
Carbon dioxide	Air	$0.01\cdot10^{-9}$	$-7.08\cdot10^{-5}$	$0.01\cdot10^{-9}$	$-6.72\cdot10^{-5}$	$2.32\cdot10^{-4}$	$-7.08\cdot10^{-5}$	$3.78\cdot10^{-4}$	$-2.66\cdot10^{-5}$
Carbon dioxide, biogenic	Air	$7.01\cdot10^{-6}$	x	$1.34\cdot10^{-5}$	x	x	x	$7.01\cdot10^{-7}$	x
Carbon dioxide, fossil	Air	$6.69\cdot10^{-4}$	x	$1.54\cdot10^{-3}$	x	x	x	$6.69\cdot10^{-5}$	x
Carbon dioxide, in air	Raw	$-6.53\cdot10^{-6}$	x	$-1.91\cdot10^{-5}$	x	x	x	$-6.53\cdot10^{-7}$	x
Carbon monoxide, fossil	Air	$1.29\cdot10^{-6}$	x	$2.15\cdot10^{-6}$	x	x	x	x	x
Dinitrogen monoxide	Air	$2.84\cdot10^{-4}$	$7.44\cdot10^{-7}$	$4.89\cdot10^{-5}$	$7.07\cdot10^{-7}$	$0.01\cdot10^{-9}$	$7.44\cdot10^{-7}$	$2.84\cdot10^{-5}$	$7.00\cdot10^{-7}$
Methane	Air	x	x	x	x	$0.01\cdot10^{-9}$	$3.64\cdot10^{-7}$	$3.01\cdot10^{-5}$	$-4.98\cdot10^{-6}$
Methane, bromotrifluoro-, Halon 1301	Air	x	x	x	x	$0.01\cdot10^{-9}$	$2.67\cdot10^{-7}$	x	x
Methane, dichloro-, HCC-30	Air	x	x	x	x	$7.13\cdot10^{-7}$	x	x	x
Methane, fossil	Air	$4.97\cdot10^{-5}$	x	$1.47\cdot10^{-4}$	x	x	x	$4.97\cdot10^{-6}$	x
Remaining substances	x	$1.01\cdot10^{-6}$	$5.35\cdot10^{-7}$	$2.53\cdot10^{-6}$	$5.08\cdot10^{-7}$	$1.13\cdot10^{-7}$	$-9.67\cdot10^{-8}$	$8.97\cdot10^{-7}$	$-5.44\cdot10^{-7}$
**TOTAL**		**$1.01\cdot10^{-3}$**	**$-6.95\cdot10^{-5}$**	**$1.74\cdot10^{-3}$**	**$-6.60\cdot10^{-5}$**	**$2.32\cdot10^{-4}$**	**$-6.95\cdot10^{-5}$**	**$5.09\cdot10^{-4}$**	**$-3.14\cdot10^{-5}$**

Table S8. Characterization results of environmental consequences for compounds causing climate change, present in selected post-production waste of wind power plant blades – part 2 [unit: DALY per 1 Mg].

SUBSTANCE	COMPA-RTMENT	SPIRAL HOSES WITH RESIN		VACUUM BAG FILM		INFUSION MATERIALS RESIDUES		SURPLUS MATERIALS	
		LIFE CYCLE	RECY-CLING	LIFE CYCLE	RECY-CLING	LIFE CYCLE	RECY-CLING	LIFE CYCLE	RECY-CLING
Carbon dioxide	Air	$3.13\cdot10^{-4}$	$-5.60\cdot10^{-5}$	$3.66\cdot10^{-4}$	$-7.08\cdot10^{-6}$	$3.14\cdot10^{-4}$	$-5.88\cdot10^{-5}$	$1.94\cdot10^{-4}$	$-4.13\cdot10^{-5}$
Carbon dioxide, biogenic	Air	x	x	x	x	$7.93\cdot10^{-7}$	x	$2.21\cdot10^{-6}$	x
Carbon dioxide, fossil	Air	x	x	x	x	$2.28\cdot10^{-5}$	x	$2.45\cdot10^{-4}$	x
Carbon dioxide, in air	Raw	x	x	x	x	$-7.62\cdot10^{-7}$	x	$-3.11\cdot10^{-6}$	x
Carbon monoxide, fossil	Air	x	x	x	x	x	x	x	x
Dinitrogen monoxide	Air	$5.63\cdot10^{-7}$	$7.29\cdot10^{-7}$	$3.56\cdot10^{-6}$	$7.44\cdot10^{-8}$	$1.28\cdot10^{-6}$	$7.20\cdot10^{-7}$	$7.72\cdot10^{-6}$	$7.44\cdot10^{-7}$
Ethane, 1,2-dichloro-1,1,2,2-tetrafluoro-, CFC-114	Air	x	x	$4.38\cdot10^{-7}$	x	x	x	x	x
Ethane, 1,1,1,2-tetrafluoro-, HFC-134a	Air	x	x	x	x	$1.89\cdot10^{-5}$	x	x	x
Methane	Air	$1.02\cdot10^{-5}$	$-1.42\cdot10^{-6}$	$1.20\cdot10^{-5}$	$3.64\cdot10^{-8}$	$7.01\cdot10^{-6}$	$-7.99\cdot10^{-7}$	$0.01\cdot10^{-9}$	$1.56\cdot10^{-6}$
Methane, bromotrifluoro-, Halon 1301	Air	x	x	$-6.39\cdot10^{-7}$	$2.67\cdot10^{-8}$	x	x	x	x
Methane, chlorodifluoro-, HCFC-22	Air	x	x	x	x	$2.78\cdot10^{-4}$	x	x	x
Methane, dichloro-, HCC-30	Air	$5.00\cdot10^{-7}$	x	x	x	x	x	x	x
Methane, dichlorodifluoro-, CFC-12	Air	x	x	x	x	$3.23\cdot10^{-5}$	x	x	x
Methane, fossil	Air	x	x	x	x	$1.22\cdot10^{-6}$	x	$2.33\cdot10^{-5}$	x
Methane, tetrachloro-, CFC-10	Air	$-8.76\cdot10^{-6}$	x	x	x	$-2.51\cdot10^{-5}$	x	x	x
Methane, trifluoro-, HFC-23	Air	x	x	x	x	$1.65\cdot10^{-4}$	x	x	x
Remaining substances	x	$7.16\cdot10^{-8}$	$-6.76\cdot10^{-8}$	$2.76\cdot10^{-7}$	$-9.67\cdot10^{-9}$	$6.11\cdot10^{-7}$	$9.67\cdot10^{-9}$	$1.22\cdot10^{-6}$	$2.21\cdot10^{-7}$
**TOTAL**		**$3.16\cdot10^{-4}$**	**$-5.68\cdot10^{-5}$**	**$3.82\cdot10^{-4}$**	**$-6.95\cdot10^{-6}$**	**$8.16\cdot10^{-4}$**	**$-5.89\cdot10^{-5}$**	**$4.70\cdot10^{-4}$**	**$-3.88\cdot10^{-5}$**

Table S9. Characterization results of environmental consequences for radioactive substances present in selected post-production waste of wind power plant blades – part 1 [unit: DALY per 1 Mg].

SUBSTANCE	COMPA-RTMENT	FIBERGLASS MAT		ROVING FABRIC		RESIN DISCS		DISTRIBUTION HOSES	
		LIFE CYCLE	RECY-CLING	LIFE CYCLE	RECY-CLING	LIFE CYCLE	RECY-CLING	LIFE CYCLE	RECY-CLING
^{14}Carbon	Air	$6.20\cdot10^{-6}$	x	$4.27\cdot10^{-6}$	x	x	x	$6.20\cdot10^{-7}$	x
^{129}Iodine	Air	$2.80\cdot10^{-8}$	x	$1.94\cdot10^{-8}$	x	x	x	$2.80\cdot10^{-9}$	x
^{222}Radon	Air	$1.29\cdot10^{-5}$	x	$8.87\cdot10^{-6}$	x	x	x	$1.29\cdot10^{-6}$	x

Remaining substances	x	$2.58 \cdot 10^{-8}$	x	$1.73 \cdot 10^{-8}$	x	x	x	$2.58 \cdot 10^{-9}$	x
**TOTAL**		**$1.91 \cdot 10^{-5}$**	**x**	**$1.32 \cdot 10^{-5}$**	**x**	**x**	**x**	**$1.91 \cdot 10^{-6}$**	**x**

Table S10. Characterization results of environmental consequences for radioactive substances present in selected post-production waste of wind power plant blades – part 2 [unit: DALY per 1 Mg].

SUBSTANCE	COMPA-RTMENT	SPIRAL HOSES WITH RESIN		VACUUM BAG FILM		INFUSION MATERIALS RESIDUES		SURPLUS MATERIALS	
		LIFE CYCLE	RECY-CLING	LIFE CYCLE	RECY-CLING	LIFE CYCLE	RECY-CLING	LIFE CYCLE	RECY-CLING
^{14}Carbon	Air	$4.56 \cdot 10^{-7}$	x	$3.12 \cdot 10^{-6}$	x	$1.20 \cdot 10^{-6}$	x	$6.79 \cdot 10^{-7}$	x
^{134}Cesium	Water	$2.55 \cdot 10^{-8}$	x	$1.75 \cdot 10^{-7}$	x	$5.12 \cdot 10^{-8}$	x	x	x
^{137}Cesium	Water	$2.85 \cdot 10^{-7}$	x	$1.95 \cdot 10^{-6}$	x	$5.72 \cdot 10^{-7}$	x	x	x
^{60}Cobalt	Water	$3.46 \cdot 10^{-8}$	x	$2.37 \cdot 10^{-7}$	x	$6.96 \cdot 10^{-8}$	x	x	x
^{129}Iodine	Air	$7.25 \cdot 10^{-9}$	x	$4.97 \cdot 10^{-8}$	x	$1.58 \cdot 10^{-8}$	x	$3.09 \cdot 10^{-9}$	x
^{85}Krypton	Air	$1.86 \cdot 10^{-8}$	x	$1.27 \cdot 10^{-7}$	x	$3.73 \cdot 10^{-8}$	x	x	x
^{226}Radium	Water	$8.58 \cdot 10^{-9}$	x	$5.85 \cdot 10^{-8}$	x	$1.75 \cdot 10^{-8}$	x	x	x
^{222}Radon	Air	$4.63 \cdot 10^{-6}$	x	$3.17 \cdot 10^{-5}$	x	$9.88 \cdot 10^{-6}$	x	$1.41 \cdot 10^{-6}$	x
Remaining substances	x	$4.73 \cdot 10^{-9}$	x	$3.24 \cdot 10^{-8}$	x	$1.01 \cdot 10^{-8}$	x	$2.77 \cdot 10^{-9}$	x
**TOTAL**		**$5.47 \cdot 10^{-6}$**	**x**	**$3.74 \cdot 10^{-5}$**	**x**	**$1.18 \cdot 10^{-5}$**	**x**	**$2.10 \cdot 10^{-6}$**	**x**

Table S11. Characterization results of environmental consequences for compounds causing an increase in the ozone hole, present in selected post-production waste of wind power plant blades – part 1 [unit: DALY per 1 Mg].

SUBSTANCE	COMPA-RTMENT	FIBERGLASS MAT		ROVING FABRIC		RESIN DISCS		DISTRIBUTION HOSES	
		LIFE CYCLE	RECY-CLING	LIFE CYCLE	RECY-CLING	LIFE CYCLE	RECY-CLING	LIFE CYCLE	RECY-CLING
Ethane, 1,2-dichloro-1,1,2,2-tetrafluoro-, CFC-114	Air	$1.08 \cdot 10^{-8}$	x	$7.36 \cdot 10^{-9}$	x	x	x	$1.08 \cdot 10^{-9}$	x
Methane, bromochlorodifluoro-, Halon 1211	Air	$1.55 \cdot 10^{-7}$	x	$6.34 \cdot 10^{-8}$	x	x	x	$1.55 \cdot 10^{-8}$	x
Methane, bromotrifluoro-, Halon 1301	Air	$5.12 \cdot 10^{-8}$	$-4.74 \cdot 10^{-7}$	$2.54 \cdot 10^{-8}$	$-4.50 \cdot 10^{-7}$	$0.02 \cdot 10^{-9}$	$-4.74 \cdot 10^{-7}$	$5.12 \cdot 10^{-9}$	$-8.62 \cdot 10^{-8}$
Methane, chlorodifluoro-, HCFC-22	Air	$4.48 \cdot 10^{-9}$	x	$1.96 \cdot 10^{-9}$	x	x	x	$4.48 \cdot 10^{-10}$	x
Methane, dichlorodifluoro-, CFC-12	Air	$1.44 \cdot 10^{-9}$	x	$6.06 \cdot 10^{-7}$	x	x	x	$1.44 \cdot 10^{-10}$	x
Methane, monochloro-, R-40	Air	$4.43 \cdot 10^{-10}$	x	$2.48 \cdot 10^{-8}$	x	x	x	$4.43 \cdot 10^{-11}$	x
Methane, tetrachloro-, CFC-10	Air	$3.81 \cdot 10^{-7}$	x	$5.02 \cdot 10^{-9}$	x	x	x	$3.81 \cdot 10^{-8}$	x
Remaining substances	x	$3.40 \cdot 10^{-13}$	$0.00 \cdot 10^{0}$	$6.12 \cdot 10^{-13}$	$0.00 \cdot 10^{0}$	x	$0.00 \cdot 10^{0}$	$3.40 \cdot 10^{-14}$	$0.00 \cdot 10^{0}$
**TOTAL**		**$6.04 \cdot 10^{-7}$**	**$-4.74 \cdot 10^{-7}$**	**$7.34 \cdot 10^{-7}$**	**$-4.50 \cdot 10^{-7}$**	**$0.02 \cdot 10^{-9}$**	**$-4.74 \cdot 10^{-7}$**	**$6.04 \cdot 10^{-8}$**	**$-8.62 \cdot 10^{-8}$**

Table S12. Characterization results of environmental consequences for compounds causing an increase in the ozone hole, present in selected post-production waste of wind power plant blades – part 2 [unit: DALY per 1 Mg].

SUBSTANCE	COMPA-RTMENT	SPIRAL HOSES WITH RESIN		VACUUM BAG FILM		INFUSION MATERIALS RESIDUES		SURPLUS MATERIALS	
		LIFE CYCLE	RECY-CLING	LIFE CYCLE	RECY-CLING	LIFE CYCLE	RECY-CLING	LIFE CYCLE	RECY-CLING
Ethane, 1,2-dichloro-1,1,2,2-tetrafluoro-, CFC-114	Air	x	x	$1.80\cdot10^{-7}$	x	x	x	$1.17\cdot10^{-9}$	x
Methane, bromochlorodifluoro-, Halon 1211	Air	x	x	x	x	x	x	$1.03\cdot10^{-8}$	x
Methane, bromotrifluoro-, Halon 1301	Air	$5.21\cdot10^{-7}$	$-3.45\cdot10^{-7}$	$1.13\cdot10^{-6}$	$-4.74\cdot10^{-8}$	$1.05\cdot10^{-6}$	$-3.80\cdot10^{-7}$	$4.11\cdot10^{-9}$	$-5.43\cdot10^{-7}$
Methane, chlorodifluoro-, HCFC-22	Air	x	x	x	x	$4.18\cdot10^{-5}$	x	x	x
Methane, dichlorodifluoro-, CFC-12	Air	x	x	$1.41\cdot10^{-9}$	x	$1.99\cdot10^{-5}$	x	$9.89\cdot10^{-8}$	x
Methane, monochloro-, R-40	Air	x	x	x	x	x	x	$4.95\cdot10^{-9}$	x
Methane, tetrachloro-, CFC-10	Air	$4.25\cdot10^{-5}$	x	$6.38\cdot10^{-9}$	x	$1.21\cdot10^{-4}$	x	$8.06\cdot10^{-10}$	x
Methane, trichlorofluoro-, CFC-11	Air	x	x	$7.99\cdot10^{-9}$	x	$3.42\cdot10^{-7}$	x	x	x
Remaining substances	x	$4.45\cdot10^{-8}$	$0.00\cdot10^{0}$	$1.46\cdot10^{-9}$	$0.00\cdot10^{0}$	$1.02\cdot10^{-7}$	$0.00\cdot10^{0}$	$3.19\cdot10^{-10}$	$0.00\cdot10^{0}$
**TOTAL**		$\mathbf{4.30\cdot10^{-5}}$	$\mathbf{-3.45\cdot10^{-7}}$	$\mathbf{1.33\cdot10^{-6}}$	$\mathbf{-4.74\cdot10^{-8}}$	$\mathbf{1.85\cdot10^{-4}}$	$\mathbf{-3.80\cdot10^{-7}}$	$\mathbf{1.21\cdot10^{-7}}$	$\mathbf{-5.43\cdot10^{-7}}$

Table S13. Characterization results of environmental consequences for eco toxic compounds present in selected post-production waste of wind power plant blades – part 1 [unit: PAF·m²/yr. per 1 Mg].

SUBSTANCE	COMPA-RTMENT	FIBERGLASS MAT		ROVING FABRIC		RESIN DISCS		DISTRIBUTION HOSES	
		LIFE CYCLE	RECY-CLING	LIFE CYCLE	RECY-CLING	LIFE CYCLE	RECY-CLING	LIFE CYCLE	RECY-CLING
Arsenic	Air	$7.91\cdot10^{1}$	x	x	x	x	x	$7.91\cdot10^{0}$	x
Cadmium	Air	$4.31\cdot10^{2}$	$1.89\cdot10^{0}$	$2.06\cdot10^{0}$	$1.80\cdot10^{0}$	$0.01\cdot10^{-9}$	$1.89\cdot10^{0}$	$4.31\cdot10^{1}$	$2.05\cdot10^{0}$
Cadmium, ion	Water	x	x	$1.71\cdot10^{0}$	$1.02\cdot10^{-1}$	x	x	x	x
Chromium	Air	$7.17\cdot10^{1}$	x	$4.30\cdot10^{1}$	x	x	x	$7.17\cdot10^{0}$	x
Chromium	Water	$0.01\cdot10^{-9}$	$3.45\cdot10^{0}$	$0.01\cdot10^{-9}$	$3.27\cdot10^{0}$	$0.01\cdot10^{-9}$	$3.45\cdot10^{0}$	$0.01\cdot10^{-9}$	$9.43\cdot10^{-1}$
Chromium VI	Water	$3.57\cdot10^{0}$	x	$1.57\cdot10^{1}$	x	x	x	x	x
Copper	Air	$1.40\cdot10^{1}$	x	$2.91\cdot10^{0}$	x	x	x	$1.40\cdot10^{0}$	x
Copper	Soil	x	x	$7.41\cdot10^{-1}$	x	x	x	x	x
Copper, ion	Water	$1.07\cdot10^{1}$	$3.65\cdot10^{0}$	$5.90\cdot10^{1}$	$3.47\cdot10^{0}$	$0.01\cdot10^{-9}$	$3.65\cdot10^{0}$	$1.07\cdot10^{0}$	$1.03\cdot10^{0}$
Lead	Air	$7.16\cdot10^{0}$	$6.82\cdot10^{0}$	$4.15\cdot10^{0}$	$6.48\cdot10^{0}$	$0.01\cdot10^{-9}$	$6.82\cdot10^{0}$	$7.16\cdot10^{-1}$	$6.16\cdot10^{0}$
Lead	Water	x	x	$5.29\cdot10^{-1}$	$2.28\cdot10^{-1}$	x	x	x	x
Mercury	Air	$2.06\cdot10^{0}$	$1.24\cdot10^{-1}$	$6.12\cdot10^{-1}$	$1.18\cdot10^{-1}$	x	x	x	x

Metallic ions, unspecified	Water	x	x	x	x	$6.96\cdot10^{-2}$	$-5.70\cdot10^{-1}$	x	x
Metals, unspecified	Air	$0.01\cdot10^{-9}$	$2.07\cdot10^{2}$	$0.01\cdot10^{-9}$	$1.96\cdot10^{2}$	$0.01\cdot10^{-9}$	$2.07\cdot10^{2}$	$6.89\cdot10^{0}$	$2.03\cdot10^{2}$
Nickel	Air	$2.87\cdot10^{2}$	$9.98\cdot10^{1}$	$1.68\cdot10^{2}$	$9.48\cdot10^{1}$	$0.01\cdot10^{-9}$	$9.98\cdot10^{1}$	$2.87\cdot10^{1}$	$1.08\cdot10^{2}$
Nickel, ion	Water	$6.66\cdot10^{0}$	$3.43\cdot10^{0}$	$3.16\cdot10^{1}$	$3.26\cdot10^{0}$	$0.01\cdot10^{-9}$	$3.43\cdot10^{0}$	$6.66\cdot10^{-1}$	$8.86\cdot10^{-1}$
Zinc	Air	$3.20\cdot10^{1}$	$5.24\cdot10^{0}$	$9.11\cdot10^{0}$	$4.98\cdot10^{0}$	$0.01\cdot10^{-9}$	$5.24\cdot10^{0}$	$3.20\cdot10^{0}$	$7.35\cdot10^{0}$
Zinc	Soil	$6.40\cdot10^{0}$	x	$6.45\cdot10^{0}$	x	x	x	$6.40\cdot10^{-1}$	x
Zinc, ion	Water	$1.31\cdot10^{0}$	$9.75\cdot10^{-1}$	$6.81\cdot10^{0}$	$9.26\cdot10^{-1}$	$0.01\cdot10^{-9}$	$9.75\cdot10^{-1}$	x	x
Remaining substances	x	$2.79\cdot10^{0}$	$-9.83\cdot10^{-2}$	$1.17\cdot10^{0}$	$-4.23\cdot10^{-1}$	$0.00\cdot10^{0}$	$5.96\cdot10^{-1}$	$1.19\cdot10^{0}$	$2.62\cdot10^{-1}$
**TOTAL**		**$9.55\cdot10^{2}$**	**$3.32\cdot10^{2}$**	**$3.53\cdot10^{2}$**	**$3.15\cdot10^{2}$**	**$6.96\cdot10^{-2}$**	**$3.32\cdot10^{2}$**	**$1.03\cdot10^{2}$**	**$3.30\cdot10^{2}$**

Table S14. Characterization results of environmental consequences for eco toxic compounds present in selected post-production waste of wind power plant blades – part 2 [unit: PAF·m²/yr. per 1 Mg].

SUBSTANCE	COMPA-RTMENT	SPIRAL HOSES WITH RESIN		VACUUM BAG FILM		INFUSION MATERIALS RESIDUES		SURPLUS MATERIALS	
		LIFE CYCLE	RECY-CLING	LIFE CYCLE	RECY-CLING	LIFE CYCLE	RECY-CLING	LIFE CYCLE	RECY-CLING
Arsenic	Air	x	x	$6.90\cdot10^{-1}$	x	x	x	x	x
Cadmium	Air	$3.20\cdot10^{0}$	$1.94\cdot10^{0}$	$1.36\cdot10^{1}$	$1.89\cdot10^{-1}$	$6.82\cdot10^{0}$	$1.89\cdot10^{0}$	$3.44\cdot10^{-1}$	$1.81\cdot10^{0}$
Cadmium, ion	Water	x	x	x	x	x	x	x	x
Chromium	Air	$1.28\cdot10^{0}$	x	$7.00\cdot10^{0}$	x	$2.76\cdot10^{0}$	x	$6.79\cdot10^{0}$	x
Chromium	Water	$0.01\cdot10^{-9}$	$2.61\cdot10^{0}$	$3.18\cdot10^{0}$	$3.45\cdot10^{-1}$	$3.80\cdot10^{-3}$	$2.83\cdot10^{0}$	$0.01\cdot10^{-9}$	$3.55\cdot10^{0}$
Chromium	Soil	$1.33\cdot10^{0}$	x	$3.01\cdot10^{0}$	x	$2.67\cdot10^{0}$	x	x	x
Chromium, ion	Water	$5.01\cdot10^{-1}$	x	x	x	$1.01\cdot10^{0}$	x	x	x
Chromium VI	Water	x	x	x	x	x	x	$2.49\cdot10^{0}$	x
Copper	Air	$1.32\cdot10^{0}$	x	$7.07\cdot10^{0}$	x	$2.97\cdot10^{0}$	x	$4.87\cdot10^{-1}$	x
Copper	Soil	x	x	x	x	x	x	x	x
Copper, ion	Water	$5.06\cdot10^{-1}$	$2.78\cdot10^{0}$	$3.37\cdot10^{0}$	$3.65\cdot10^{-1}$	$1.80\cdot10^{0}$	$3.00\cdot10^{0}$	$9.42\cdot10^{0}$	$3.79\cdot10^{0}$
Lead	Air	$2.15\cdot10^{0}$	$6.60\cdot10^{0}$	$1.29\cdot10^{1}$	$6.82\cdot10^{-1}$	$4.79\cdot10^{0}$	$6.54\cdot10^{0}$	$6.97\cdot10^{-1}$	$6.75\cdot10^{0}$
Mercury	Air	$3.85\cdot10^{-1}$	$1.24\cdot10^{-1}$	$6.07\cdot10^{-1}$	$1.24\cdot10^{-2}$	$9.28\cdot10^{-1}$	$1.23\cdot10^{-1}$	x	x
Metals, unspecified	Air	$1.53\cdot10^{0}$	$2.05\cdot10^{2}$	$1.28\cdot10^{0}$	$2.07\cdot10^{1}$	$2.81\cdot10^{0}$	$2.02\cdot10^{2}$	$4.98\cdot10^{0}$	$2.01\cdot10^{2}$
Nickel	Air	$5.48\cdot10^{1}$	$1.03\cdot10^{2}$	$2.42\cdot10^{2}$	$9.98\cdot10^{0}$	$1.16\cdot10^{2}$	$9.97\cdot10^{1}$	$2.66\cdot10^{1}$	$9.97\cdot10^{1}$
Nickel, ion	Water	$5.04\cdot10^{-1}$	$2.59\cdot10^{0}$	$3.29\cdot10^{0}$	$3.43\cdot10^{-1}$	$1.35\cdot10^{0}$	$2.80\cdot10^{0}$	$5.01\cdot10^{0}$	$3.56\cdot10^{0}$
Zinc	Air	$3.21\cdot10^{0}$	$5.94\cdot10^{0}$	$1.93\cdot10^{1}$	$5.24\cdot10^{-1}$	$7.33\cdot10^{0}$	$5.59\cdot10^{0}$	$1.48\cdot10^{0}$	$4.60\cdot10^{0}$
Zinc	Soil	$3.01\cdot10^{0}$	x	$6.81\cdot10^{0}$	x	$6.43\cdot10^{0}$	x	$1.03\cdot10^{0}$	x
Zinc, ion	Water	$1.32\cdot10^{-1}$	$7.33\cdot10^{-1}$	$8.04\cdot10^{-1}$	$9.75\cdot10^{-2}$	$3.58\cdot10^{-1}$	$7.94\cdot10^{-1}$	$1.08\cdot10^{0}$	$9.43\cdot10^{-1}$
Remaining substances	x	$4.46\cdot10^{-1}$	$-1.03\cdot10^{-1}$	$8.57\cdot10^{-1}$	$-9.83\cdot10^{-3}$	$1.15\cdot10^{0}$	$-9.92\cdot10^{-2}$	$1.22\cdot10^{0}$	$5.88\cdot10^{-2}$
**TOTAL**		**$7.43\cdot10^{1}$**	**$3.31\cdot10^{2}$**	**$3.26\cdot10^{2}$**	**$3.32\cdot10^{1}$**	**$1.59\cdot10^{2}$**	**$3.25\cdot10^{2}$**	**$6.17\cdot10^{1}$**	**$3.26\cdot10^{2}$**

Table S15. Characterization results of environmental consequences for compounds causing acidification or eutrophication present in selected post-production waste of wind power plant blades – part 1 [unit: PDF·m²/yr. per 1 Mg].

SUBSTANCE	COMPA-RTMENT	FIBERGLASS MAT		ROVING FABRIC		RESIN DISCS		DISTRIBUTION HOSES	
		LIFE CYCLE	RECY-CLING	LIFE CYCLE	RECY-CLING	LIFE CYCLE	RECY-CLING	LIFE CYCLE	RECY-CLING
Ammonia	Air	$1.54\cdot10^{0}$	$5.71\cdot10^{-2}$	$5.44\cdot10^{-1}$	$5.43\cdot10^{-2}$	x	x	$1.54\cdot10^{-1}$	$5.08\cdot10^{-2}$

Nitric oxide	Air	x	x	x	x	$1.42\cdot10^{-1}$	x	x	x
Nitrogen oxides	Air	$4.57\cdot10^{1}$	$-3.44\cdot10^{1}$	$7.72\cdot10^{1}$	$-3.26\cdot10^{1}$	$9.69\cdot10^{1}$	$-3.44\cdot10^{1}$	$5.39\cdot10^{1}$	$-5.75\cdot10^{1}$
Sulfate	Air	$8.64\cdot10^{-1}$	x	x	x	x	x	$8.64\cdot10^{-2}$	x
Sulfur dioxide	Air	$1.12\cdot10^{1}$	x	$2.06\cdot10^{1}$	x	$1.43\cdot10^{0}$	x	$1.12\cdot10^{0}$	x
Sulfur oxides	Air	$0.01\cdot10^{-9}$	$2.36\cdot10^{0}$	x	$2.24\cdot10^{0}$	$2.44\cdot10^{-1}$	$2.36\cdot10^{0}$	$7.68\cdot10^{0}$	$-2.70\cdot10^{0}$
Remaining substances	x	$8.45\cdot10^{-6}$	$-3.55\cdot10^{-15}$	$3.14\cdot10^{-2}$	$0.00\cdot10^{0}$	$7.12\cdot10^{-3}$	$5.71\cdot10^{-2}$	$8.45\cdot10^{-7}$	$-7.11\cdot10^{-15}$
**TOTAL**		**$5.93\cdot10^{1}$**	**$-3.19\cdot10^{1}$**	**$9.84\cdot10^{1}$**	**$-3.03\cdot10^{1}$**	**$9.88\cdot10^{1}$**	**$-3.19\cdot10^{1}$**	**$6.30\cdot10^{1}$**	**$-6.01\cdot10^{1}$**

Table S16. Characterization results of environmental consequences for compounds causing acidification or eutrophication present in selected post-production waste of wind power plant blades – part 2 [unit: PDF·m^2/yr. per 1 Mg].

SUBSTANCE	COMPA-RTMENT	SPIRAL HOSES WITH RESIN		VACUUM BAG FILM		INFUSION MATERIALS RESIDUES		SURPLUS MATERIALS	
		LIFE CYCLE	RECY-CLING	LIFE CYCLE	RECY-CLING	LIFE CYCLE	RECY-CLING	LIFE CYCLE	RECY-CLING
Ammonia	Air	$1.40\cdot10^{-2}$	$5.50\cdot10^{-2}$	$6.26\cdot10^{-2}$	$5.71\cdot10^{-3}$	$4.33\cdot10^{-2}$	$5.46\cdot10^{-2}$	$8.99\cdot10^{-2}$	$5.75\cdot10^{-2}$
Nitric oxide	Air	$9.92\cdot10^{-2}$	x	x	x	$7.79\cdot10^{-2}$	x	$6.38\cdot10^{-2}$	x
Nitrogen oxides	Air	$8.26\cdot10^{1}$	$-4.21\cdot10^{1}$	$2.39\cdot10^{1}$	$-3.44\cdot10^{0}$	$7.71\cdot10^{1}$	$-3.88\cdot10^{1}$	$7.83\cdot10^{1}$	$-3.21\cdot10^{1}$
Sulfate	Air	x	x	x	x	$2.59\cdot10^{-1}$	x	x	x
Sulfur dioxide	Air	$9.99\cdot10^{-1}$	x	x	x	$1.81\cdot10^{0}$	x	$3.91\cdot10^{0}$	x
Sulfur oxides	Air	$3.85\cdot10^{0}$	$6.71\cdot10^{-1}$	$1.12\cdot10^{1}$	$2.36\cdot10^{-1}$	$6.23\cdot10^{0}$	$1.15\cdot10^{0}$	$4.55\cdot10^{0}$	$9.09\cdot10^{-1}$
Remaining substances	x	$0.00\cdot10^{0}$	$7.11\cdot10^{-15}$	$0.00\cdot10^{0}$	$0.00\cdot10^{0}$	$2.76\cdot10^{-7}$	$-7.11\cdot10^{-15}$	$5.14\cdot10^{-3}$	$0.00\cdot10^{0}$
**TOTAL**		**$8.75\cdot10^{1}$**	**$-4.13\cdot10^{1}$**	**$3.51\cdot10^{1}$**	**$-3.19\cdot10^{0}$**	**$8.56\cdot10^{1}$**	**$-3.76\cdot10^{1}$**	**$8.69\cdot10^{1}$**	**$-3.11\cdot10^{1}$**

Table S17. Characterization results of environmental consequences for processes related to land use, present in selected post-production waste of wind power plants blades – part 1 [unit: PDF·m^2/yr. per 1 Mg].

PROCESS	COMPA-RTMENT	FIBERGLASS MAT		ROVING FABRIC		RESIN DISCS		DISTRIBUTION HOSES	
		LIFE CYCLE	RECY-CLING	LIFE CYCLE	RECY-CLING	LIFE CYCLE	RECY-CLING	LIFE CYCLE	RECY-CLING
Occupation, arable, non-irrigated	Raw	$7.34\cdot10^{-3}$	x	$9.71\cdot10^{-2}$	x	x	x	x	x
Occupation, construction site	Raw	$1.78\cdot10^{-2}$	x	$1.00\cdot10^{-1}$	x	x	x	x	x
Occupation, dump site	Raw	$1.49\cdot10^{0}$	x	$1.10\cdot10^{0}$	x	x	x	$1.49\cdot10^{-1}$	x
Occupation, forest, intensive	Raw	$1.63\cdot10^{-2}$	x	$7.02\cdot10^{-2}$	x	x	x	x	x
Occupation, forest, intensive, normal	Raw	$4.99\cdot10^{-1}$	x	$1.35\cdot10^{1}$	x	x	x	$4.99\cdot10^{-2}$	x
Occupation, industrial area	Raw	$4.38\cdot10^{-1}$	x	$2.50\cdot10^{-1}$	x	$2.76\cdot10^{1}$	x	$3.03\cdot10^{1}$	x
Occupation, industrial area, vegetation	Raw	x	x	$3.06\cdot10^{-2}$	x	x	x	x	x
Occupation, mineral extraction site	Raw	$2.90\cdot10^{0}$	x	$5.86\cdot10^{-1}$	x	x	x	$2.90\cdot10^{-1}$	x
Occupation, traffic area, road embankment	Raw	$3.97\cdot10^{-2}$	x	$1.03\cdot10^{0}$	x	x	x	x	x
Transformation, from arable	Raw	$-1.07\cdot10^{-1}$	x	$-5.37\cdot10^{0}$	x	x	x	x	x
Transformation, from arable, non-irrigated	Raw	$-4.04\cdot10^{-1}$	x	x	x	x	x	$-4.04\cdot10^{-2}$	x

Transformation, from arable, non-irrigated, fallow	Raw	$-3.41\cdot10^{-2}$	x	x	x	x	x	x	x
Transformation, from dump site, inert material landfill	Raw	$-1.46\cdot10^{-1}$	x	$-3.91\cdot10^{-1}$	x	x	x	x	x
Transformation, from dump site, residual material landfill	Raw	$-6.52\cdot10^{-2}$	x	$-1.42\cdot10^{-1}$	x	x	x	x	x
Transformation, from forest	Raw	$-7.81\cdot10^{-2}$	x	x	x	x	x	x	x
Transformation, from forest, extensive	Raw	$-1.22\cdot10^{-1}$	x	$-3.38\cdot10^{0}$	x	x	x	x	x
Transformation, from industrial area	Raw	$-3.19\cdot10^{-2}$	x	$-2.20\cdot10^{-2}$	x	x	x	x	x
Transformation, from mineral extraction site	Raw	$-1.79\cdot10^{0}$	x	$-3.42\cdot10^{-1}$	x	x	x	$-1.79\cdot10^{-1}$	x
Transformation, from pasture and meadow	Raw	$-4.19\cdot10^{-1}$	x	$-7.82\cdot10^{-1}$	x	x	x	$-4.19\cdot10^{-2}$	x
Transformation, from sea and ocean	Raw	x	x	$-2.92\cdot10^{-2}$	x	x	x	x	x
Transformation, from shrub land, sclerophyllous	Raw	$-4.49\cdot10^{-2}$	x	$-8.29\cdot10^{-2}$	x	x	x	x	x
Transformation, from unknown	Raw	$-4.13\cdot10^{0}$	x	$-1.28\cdot10^{0}$	x	x	x	$-4.13\cdot10^{-1}$	x
Transformation, to arable	Raw	$7.74\cdot10^{-1}$	x	$4.10\cdot10^{-1}$	x	x	x	$7.74\cdot10^{-2}$	x
Transformation, to arable, non-irrigated	Raw	$4.04\cdot10^{-1}$	x	$5.37\cdot10^{0}$	x	x	x	$4.04\cdot10^{-2}$	x
Transformation, to arable, non-irrigated, fallow	Raw	$7.38\cdot10^{-2}$	x	x	x	x	x	x	x
Transformation, to dump site	Raw	$3.43\cdot10^{-1}$	x	$2.05\cdot10^{-1}$	x	x	x	$3.43\cdot10^{-2}$	x
Transformation, to dump site, benthos	Raw	x	x	$2.29\cdot10^{-2}$	x	x	x	x	x
Transformation, to dump site, inert material landfill	Raw	$1.46\cdot10^{-1}$	x	$3.91\cdot10^{-1}$	x	x	x	x	x
Transformation, to dump site, residual material landfill	Raw	$6.52\cdot10^{-2}$	x	$1.42\cdot10^{-1}$	x	x	x	x	x
Transformation, to forest	Raw	$1.08\cdot10^{-1}$	x	$7.53\cdot10^{-2}$	x	x	x	x	x
Transformation, to forest, intensive, normal	Raw	$1.15\cdot10^{-1}$	x	$3.34\cdot10^{0}$	x	x	x	x	x
Transformation, to industrial area	Raw	$1.34\cdot10^{-1}$	x	$7.55\cdot10^{-2}$	x	$2.16\cdot10^{0}$	x	x	x
Transformation, to industrial area, built up	Raw	$8.19\cdot10^{-3}$	x	x	x	x	x	x	x
Transformation, to mineral extraction site	Raw	$3.15\cdot10^{0}$	x	$5.75\cdot10^{-1}$	x	x	x	$3.15\cdot10^{-1}$	x
Transformation, to shrub land, sclerophyllous	Raw	$2.79\cdot10^{-2}$	x	$7.03\cdot10^{-2}$	x	x	x	x	x
Transformation, to traffic area, road embankment	Raw	$9.08\cdot10^{-3}$	x	$2.54\cdot10^{-1}$	x	x	x	x	x
Transformation, to unknown	Raw	$8.11\cdot10^{-1}$	x	$2.99\cdot10^{-2}$	x	x	x	$8.11\cdot10^{-2}$	x
Transformation, to water bodies, artificial	Raw	$8.16\cdot10^{-1}$	x	$3.44\cdot10^{-1}$	x	x	x	$8.16\cdot10^{-2}$	x
Transformation, to water courses, artificial	Raw	$2.94\cdot10^{-1}$	x	$2.17\cdot10^{-1}$	x	x	x	x	x

Remaining substances	x	$1.04\cdot10^{-2}$	x	$7.78\cdot10^{-2}$	x	$0.00\cdot10^{0}$	x	$4.44\cdot10^{-2}$	x
**TOTAL**		**$5.32\cdot10^{0}$**	**x**	**$1.66\cdot10^{1}$**	**x**	**$2.98\cdot10^{1}$**	**x**	**$3.08\cdot10^{1}$**	**x**

Table S18. Characterization results of environmental consequences for processes related to land use, present in selected post-production waste of wind power plant blades – part 2 [unit: PDF·m²/yr. per 1 Mg].

SUBSTANCE	COMPA-RTMENT	SPIRAL HOSES WITH RESIN		VACUUM BAG FILM		INFUSION MATERIALS RESIDUES		SURPLUS MATERIALS	
		LIFE CYCLE	RECY-CLING	LIFE CYCLE	RECY-CLING	LIFE CYCLE	RECY-CLING	LIFE CYCLE	RECY-CLING
Land use II-III	Raw	$6.65\cdot10^{0}$	x	$4.58\cdot10^{1}$	x	$1.34\cdot10^{1}$	x	x	x
Land use II-IV	Raw	$5.37\cdot10^{-2}$	x	$1.34\cdot10^{0}$	x	$1.14\cdot10^{-1}$	x	x	x
Land use III-IV	Raw	x	x	$9.07\cdot10^{-1}$	x	$6.53\cdot10^{-2}$	x	x	x
Occupation, dump site	Raw	x	x	x	x	$7.07\cdot10^{-2}$	x	$1.78\cdot10^{-1}$	x
Occupation, forest, intensive, normal	Raw	x	x	x	x	$1.46\cdot10^{-1}$	x	$2.16\cdot10^{0}$	x
Occupation, industrial area	Raw	$2.60\cdot10^{1}$	x	$3.42\cdot10^{0}$	x	$2.28\cdot10^{1}$	x	$2.83\cdot10^{1}$	x
Occupation, mineral extraction site	Raw	x	x	x	x	$4.65\cdot10^{-2}$	x	$9.51\cdot10^{-2}$	x
Occupation, traffic area, road embankment	Raw	x	x	x	x	x	x	$1.65\cdot10^{-1}$	x
Transformation, from arable, non-irrigated	Raw	x	x	x	x	x	x	$-8.52\cdot10^{-1}$	x
Transformation, from dump site, inert material landfill	Raw	x	x	x	x	x	x	$-6.18\cdot10^{-2}$	x
Transformation, from forest, extensive	Raw	x	x	x	x	x	x	$-5.40\cdot10^{-1}$	x
Transformation, from mineral extraction site	Raw	x	x	x	x	x	x	$-5.58\cdot10^{-2}$	x
Transformation, from pasture and meadow	Raw	x	x	x	x	x	x	$-1.25\cdot10^{-1}$	x
Transformation, from unknown	Raw	x	x	x	x	$-1.06\cdot10^{-1}$	x	$-2.12\cdot10^{-1}$	x
Transformation, to arable	Raw	x	x	x	x	x	x	$6.51\cdot10^{-2}$	x
Transformation, to arable, non-irrigated	Raw	x	x	x	x	x	x	$8.53\cdot10^{-1}$	x
Transformation, to dump site	Raw	x	x	x	x	x	x	$3.33\cdot10^{-2}$	x
Transformation, to dump site, inert material landfill	Raw	x	x	x	x	x	x	$6.18\cdot10^{-2}$	x
Transformation, to forest, intensive, normal	Raw	x	x	x	x	x	x	$5.32\cdot10^{-1}$	x
Transformation, to industrial area	Raw	$1.51\cdot10^{0}$	x	x	x	$1.20\cdot10^{0}$	x	$9.86\cdot10^{-1}$	x
Transformation, to mineral extraction site	Raw	x	x	x	x	$4.56\cdot10^{-2}$	x	$9.56\cdot10^{-2}$	x
Transformation, to traffic area, road embankment	Raw	x	x	x	x	x	x	$4.05\cdot10^{-2}$	x
Transformation, to water bodies, artificial	Raw	x	x	x	x	x	x	$5.75\cdot10^{-2}$	x
Transformation, to water courses, artificial	Raw	x	x	x	x	x	x	$3.55\cdot10^{-2}$	x
Remaining substances	x	$2.80\cdot10^{-2}$	x	$7.10\cdot10^{-3}$	x	$8.77\cdot10^{-2}$	x	$7.32\cdot10^{-2}$	x
**TOTAL**		**$3.43\cdot10^{1}$**	**x**	**$5.15\cdot10^{1}$**	**x**	**$3.78\cdot10^{1}$**	**x**	**$3.18\cdot10^{1}$**	**x**

Table S19. Characterization results of environmental consequences for processes related to the extraction of mineral resources present in selected post-production waste of wind power plant blades – part 1 [unit: MJ surplus per 1 Mg].

SUBSTANCE	COMPA-RTMENT	FIBERGLASS MAT		ROVING FABRIC		RESIN DISCS		DISTRIBUTION HOSES	
		LIFE CYCLE	RECY-CLING	LIFE CYCLE	RECY-CLING	LIFE CYCLE	RECY-CLING	LIFE CYCLE	RECY-CLING
Aluminium, 24% in bauxite, 11% in crude ore, in ground	Raw	$1.94\cdot10^{1}$	x	$1.19\cdot10^{0}$	x	x	x	$1.94\cdot10^{0}$	x
Bauxite, in ground	Raw	$0.01\cdot10^{-9}$	$-1.35\cdot10^{-1}$	$0.01\cdot10^{-9}$	$-1.28\cdot10^{-1}$	$7.80\cdot10^{-1}$	$-1.35\cdot10^{-1}$	$1.98\cdot10^{-1}$	$-1.03\cdot10^{-1}$
Chromium, 25.5% in chromite, 11.6% in crude ore, in ground	Raw	x	x	$8.50\cdot10^{-3}$	x	x	x	x	x
Cinnabar, in ground	Raw	$1.03\cdot10^{-1}$	x	$9.68\cdot10^{-3}$	x	x	x	$1.03\cdot10^{-2}$	x
Copper, 0.99% in sulfide, Cu 0.36% and Mo $8.2\cdot10^{-3}$% in crude ore, in ground	Raw	$2.60\cdot10^{-2}$	x	$1.89\cdot10^{-1}$	x	x	x	$2.60\cdot10^{-3}$	x
Copper, 1.18% in sulfide, Cu 0.39% and Mo $8.2\cdot10^{-3}$% in crude ore, in ground	Raw	$4.60\cdot10^{-2}$	x	$6.26\cdot10^{-1}$	x	x	x	$4.60\cdot10^{-3}$	x
Copper, 1.42% in sulfide, Cu 0.81% and Mo $8.2\cdot10^{-3}$% in crude ore, in ground	Raw	x	x	$1.66\cdot10^{-1}$	x	x	x	x	x
Copper, 2.19% in sulfide, Cu 1.83% and Mo $8.2\cdot10^{-3}$% in crude ore, in ground	Raw	$6.05\cdot10^{-2}$	x	$8.24\cdot10^{-1}$	x	x	x	$6.05\cdot10^{-3}$	x
Copper, in ground	Raw	x	x	x	x	x	x	x	x
Iron, 46% in ore, 25% in crude ore, in ground	Raw	$2.13\cdot10^{-2}$	x	$1.55\cdot10^{-1}$	x	x	x	$2.13\cdot10^{-3}$	x
Iron ore, in ground	Raw	x	x	x	x	$0.01\cdot10^{-9}$	$-5.22\cdot10^{-3}$	x	x
Molybdenum, 0.010% in sulfide, Mo $8.2\cdot10^{-3}$% and Cu 1.83% in crude ore, in ground	Raw	x	x	$1.71\cdot10^{-2}$	x	x	x	x	x
Molybdenum, 0.022% in sulfide, Mo $8.2\cdot10^{-3}$% and Cu 0.36% in crude ore, in ground	Raw	x	x	$5.80\cdot10^{-2}$	x	x	x	x	x
Molybdenum, 0.025% in sulfide, Mo $8.2\cdot10^{-3}$% and Cu 0.39% in crude ore, in ground	Raw	x	x	$8.93\cdot10^{-3}$	x	x	x	x	x
Molybdenum, 0.11% in sulfide, Mo $4.1\cdot10^{-2}$% and Cu 0.36% in crude ore, in ground	Raw	$2.27\cdot10^{-2}$	x	$1.17\cdot10^{-1}$	x	x	x	$2.27\cdot10^{-3}$	x
Nickel, 1.13% in sulfide, Ni 0.76% and Cu 0.76% in crude ore, in ground	Raw	$1.05\cdot10^{-1}$	x	$3.94\cdot10^{-2}$	x	x	x	$1.05\cdot10^{-2}$	x
Nickel, 1.98% in silicates, 1.04% in crude ore, in ground	Raw	$2.93\cdot10^{-1}$	x	$1.05\cdot10^{0}$	x	x	x	$2.93\cdot10^{-2}$	x
Nickel, in ground	Raw	x	x	x	x	x	x	x	x

Tin, 79% in cassiterite, 0.1% in crude ore, in ground	Raw	$4.64\cdot10^{-2}$	x	$2.60\cdot10^{0}$	x	x	x	$4.64\cdot10^{-3}$	x
Remaining substances	x	$3.13\cdot10^{-2}$	$-5.22\cdot10^{-3}$	$3.84\cdot10^{-3}$	$-4.96\cdot10^{-3}$	$0.00\cdot10^{0}$	$0.00\cdot10^{0}$	$1.36\cdot10^{-2}$	$-9.92\cdot10^{-3}$
**TOTAL**		**$2.02\cdot10^{1}$**	**$-1.40\cdot10^{-1}$**	**$7.07\cdot10^{0}$**	**$-1.33\cdot10^{-1}$**	**$7.80\cdot10^{-1}$**	**$-1.40\cdot10^{-1}$**	**$2.23\cdot10^{0}$**	**$-1.13\cdot10^{-1}$**

Table S20. Characterization results of environmental consequences for processes related to the extraction of mineral resources present in selected post-production waste of wind power plant blades – part 2 [unit: MJ surplus per 1 Mg].

SUBSTANCE	COMPA-RTMENT	SPIRAL HOSES WITH RESIN		VACUUM BAG FILM		INFUSION MATERIALS RESIDUES		SURPLUS MATERIALS	
		LIFE CYCLE	RECY-CLING	LIFE CYCLE	RECY-CLING	LIFE CYCLE	RECY-CLING	LIFE CYCLE	RECY-CLING
Aluminium, 24% in bauxite, 11% in crude ore, in ground	Raw	x	x	x	x	$1.89\cdot10^{-3}$	x	$1.88\cdot10^{-1}$	x
Bauxite, in ground	Raw	$5.90\cdot10^{-1}$	$-1.24\cdot10^{-1}$	$3.05\cdot10^{-1}$	$-1.35\cdot10^{-2}$	$4.63\cdot10^{-1}$	$-1.26\cdot10^{-1}$	$4.29\cdot10^{-1}$	$-1.51\cdot10^{-1}$
Chromium, in ground	Raw	x	x	$1.97\cdot10^{-2}$	x	x	x	x	x
Cinnabar, in ground	Raw	x	x	x	x	$9.31\cdot10^{-3}$	x	x	x
Copper, 0.99% in sulfide, Cu 0.36% and Mo $8.2\cdot10^{-3}$% in crude ore, in ground	Raw	x	x	x	x	$2.12\cdot10^{-2}$	x	$3.43\cdot10^{-2}$	x
Copper, 1.18% in sulfide, Cu 0.39% and Mo $8.2\cdot10^{-3}$% in crude ore, in ground	Raw	x	x	x	x	$1.17\cdot10^{-1}$	x	$1.23\cdot10^{-1}$	x
Copper, 1.42% in sulfide, Cu 0.81% and Mo $8.2\cdot10^{-3}$% in crude ore, in ground	Raw	x	x	x	x	$3.10\cdot10^{-2}$	x	$3.27\cdot10^{-2}$	x
Copper, 2.19% in sulfide, Cu 1.83% and Mo $8.2\cdot10^{-3}$% in crude ore, in ground	Raw	x	x	x	x	$1.54\cdot10^{-1}$	x	$1.62\cdot10^{-1}$	x
Copper, in ground	Raw	x	x	$9.38\cdot10^{0}$	x	$2.00\cdot10^{-2}$	x	x	x
Iron, 46% in ore, 25% in crude ore, in ground	Raw	x	x	x	x	x	x	$2.46\cdot10^{-2}$	x
Iron ore, in ground	Raw	$2.32\cdot10^{-3}$	$-6.79\cdot10^{-3}$	x	x	$1.31\cdot10^{-3}$	$-6.19\cdot10^{-3}$	$3.36\cdot10^{-3}$	$-6.16\cdot10^{-3}$
Iron, in ground	Raw	$2.93\cdot10^{-3}$	x	$2.12\cdot10^{-1}$	x	$6.76\cdot10^{-3}$	x	x	x
Lead, in ground	Raw	x	x	$1.40\cdot10^{-1}$	x	x	x	x	x
Molybdenum, 0.010% in sulfide, Mo $8.2\cdot10^{-3}$% and Cu 1.83% in crude ore, in ground	Raw	x	x	x	x	$3.19\cdot10^{-3}$	x	$3.37\cdot10^{-3}$	x
Molybdenum, 0.022% in sulfide, Mo $8.2\cdot10^{-3}$% and Cu 0.36% in crude ore, in ground	Raw	x	x	x	x	x	x	$9.24\cdot10^{-3}$	x

Molybdenum, 0.025% in sulfide, Mo 8.2·10-3% and Cu 0.39% in crude ore, in ground	Raw	x	x	x	x	$1.67·10^{-3}$	x	$1.76·10^{-3}$	x
Molybdenum, 0.11% in sulfide, Mo 4.1·10-2% and Cu 0.36% in crude ore, in ground	Raw	x	x	x	x	x	x	$1.87·10^{-2}$	x
Nickel, 1.13% in sulfide, Ni 0.76% and Cu 0.76% in crude ore, in ground	Raw	x	x	x	x	x	x	$6.41·10^{-3}$	x
Nickel, 1.98% in silicates, 1.04% in crude ore, in ground	Raw	x	x	x	x	$1.76·10^{-2}$	x	$1.68·10^{-1}$	x
Nickel, in ground	Raw	$9.27·10^{-4}$	x	$3.61·10^{-1}$	x	$2.60·10^{-3}$	x	x	x
Tin, 79% in cassiterite, 0.1% in crude ore, in ground	Raw	x	x	x	x	$5.19·10^{-1}$	x	$5.20·10^{-1}$	x
Tin, in ground	Raw	x	x	$2.44·10^{-1}$	x	$1.69·10^{-3}$	x	x	x
Remaining substances	x	$6.05·10^{-5}$	$0.00·10^{0}$	$2.32·10^{-3}$	$-5.22·10^{-4}$	$4.41·10^{-3}$	$0.00·10^{0}$	$3.61·10^{-3}$	$0.00·10^{0}$
**TOTAL**		**$5.96·10^{-1}$**	**$-1.31·10^{-1}$**	**$1.07·10^{1}$**	**$-1.40·10^{-2}$**	**$1.38·10^{0}$**	**$-1.32·10^{-1}$**	**$1.73·10^{0}$**	**$-1.57·10^{-1}$**

Table S21. Characterization results of environmental consequences for processes related to the extraction of fossil fuels present in selected post-production waste of wind power plant blades – part 1 [unit: MJ surplus per 1 Mg].

SUBSTANCE	COMPA-RTMENT	FIBERGLASS MAT		ROVING FABRIC		RESIN DISCS		DISTRIBUTION HOSES	
		LIFE CYCLE	RECY-CLING	LIFE CYCLE	RECY-CLING	LIFE CYCLE	RECY-CLING	LIFE CYCLE	RECY-CLING
Coal, 18 MJ per kg, in ground	Raw	$0.01·10^{-9}$	$5.18·10^{1}$	$0.01·10^{-9}$	$4.92·10^{1}$	$0.01·10^{-9}$	$5.18·10^{1}$	$0.01·10^{-9}$	$2.25·10^{1}$
Coal, 29.3 MJ per kg, in ground	Raw	x	x	x	x	x	x	$3.06·10^{1}$	x
Coal, hard, unspecified, in ground	Raw	$5.37·10^{1}$	x	$1.40·10^{2}$	x	x	x	$5.37·10^{0}$	x
Gas, mine, off-gas, process, coal mining/m³	Raw	$1.54·10^{1}$	x	$8.73·10^{0}$	x	x	x	$1.54·10^{0}$	x
Gas, natural, 30.3 MJ per kg, in ground	Raw	x	x	x	x	$3.18·10^{3}$	x	$2.05·10^{3}$	x
Gas, natural, 35 MJ per m³, in ground	Raw	$0.01·10^{-9}$	$4.23·10^{2}$	x	$4.02·10^{2}$	$0.01·10^{-9}$	$4.23·10^{2}$	$0.01·10^{-9}$	$4.23·10^{2}$
Gas, natural, 36.6 MJ per m³, in ground	Raw	$0.01·10^{-9}$	$-2.67·10^{3}$	$0.01·10^{-9}$	$-2.53·10^{3}$	$0.01·10^{-9}$	$-2.67·10^{3}$	$0.01·10^{-9}$	$-2.05·10^{3}$
Gas, natural, feedstock, 35 MJ per m³, in ground	Raw	$0.01·10^{-9}$	$-2.93·10^{3}$	$0.01·10^{-9}$	$-2.78·10^{3}$	$0.01·10^{-9}$	$-2.93·10^{3}$	$0.01·10^{-9}$	$-1.70·10^{3}$
Gas, natural, in ground	Raw	$4.60·10^{3}$	x	$8.81·10^{3}$	x	x	x	$4.60·10^{2}$	x
Oil, crude, 42.6 MJ per kg, in ground	Raw	$0.01·10^{-9}$	$-7.23·10^{2}$	$0.01·10^{-9}$	$-6.87·10^{2}$	$0.01·10^{-9}$	$-7.23·10^{2}$	$0.01·10^{-9}$	$-2.77·10^{1}$

Oil, crude, 42.7 MJ per kg, in ground	Raw	x	x	x	x	$3.10\cdot10^{4}$	x	$2.21\cdot10^{3}$	x
Oil, crude, feedstock, 41 MJ per kg, in ground	Raw	$0.01\cdot10^{-9}$	$-2.76\cdot10^{3}$	$0.01\cdot10^{-9}$	$-2.62\cdot10^{3}$	$0.01\cdot10^{-9}$	$-2.76\cdot10^{3}$	$0.01\cdot10^{-9}$	$-2.04\cdot10^{3}$
Oil, crude, in ground	Raw	$3.15\cdot10^{3}$	x	$5.07\cdot10^{3}$	x	x	x	$3.15\cdot10^{2}$	x
Remaining substances	x	$-1.82\cdot10^{-12}$	$0.00\cdot10^{0}$	$-1.82\cdot10^{-12}$	$0.00\cdot10^{0}$	$0.00\cdot10^{0}$	$0.00\cdot10^{0}$	$-9.09\cdot10^{-13}$	$0.00\cdot10^{0}$
**TOTAL**		**$7.82\cdot10^{3}$**	**$-8.61\cdot10^{3}$**	**$1.40\cdot10^{4}$**	**$-8.18\cdot10^{3}$**	**$3.41\cdot10^{4}$**	**$-8.61\cdot10^{3}$**	**$5.07\cdot10^{3}$**	**$-5.37\cdot10^{3}$**

Table S22. Characterization results of environmental consequences for processes related to the extraction of fossil fuels present in selected post-production waste of wind power plant blades – part 2 [unit: MJ surplus per 1 Mg].

SUBSTANCE	COMPA-RTMENT	SPIRAL HOSES WITH RESIN		VACUUM BAG FILM		INFUSION MATERIALS RESIDUES		SURPLUS MATERIALS	
		LIFE CYCLE	RECY-CLING	LIFE CYCLE	RECY-CLING	LIFE CYCLE	RECY-CLING	LIFE CYCLE	RECY-CLING
Coal, 18 MJ per kg, in ground	Raw	$6.33\cdot10^{0}$	$4.20\cdot10^{1}$	$4.34\cdot10^{1}$	$5.18\cdot10^{0}$	$1.27\cdot10^{1}$	$4.43\cdot10^{1}$	x	$5.33\cdot10^{1}$
Coal, 29.3 MJ per kg, in ground	Raw	x	x	$2.77\cdot10^{0}$	x	x	x	x	x
Coal, hard, unspecified, in ground	Raw	x	x	x	x	x	x	$2.22\cdot10^{1}$	x
Gas, mine, off-gas, process, coal mining/m³	Raw	x	x	$1.23\cdot10^{1}$	x	x	x	x	x
Gas, natural, 30.3 MJ per kg, in ground	Raw	$2.68\cdot10^{3}$	x	$3.89\cdot10^{2}$	x	$2.58\cdot10^{3}$	x	$2.16\cdot10^{3}$	x
Gas, natural, 35 MJ per m³, in ground	Raw	$3.92\cdot10^{1}$	$4.23\cdot10^{2}$	$2.51\cdot10^{2}$	$4.23\cdot10^{1}$	$7.87\cdot10^{1}$	$4.14\cdot10^{2}$	$0.01\cdot10^{-9}$	$4.18\cdot10^{2}$
Gas, natural, 36.6 MJ per m³, in ground	Raw	$0.01\cdot10^{-9}$	$-2.46\cdot10^{3}$	$0.01\cdot10^{-9}$	$-2.67\cdot10^{2}$	$0.01\cdot10^{-9}$	$-2.47\cdot10^{3}$	$0.01\cdot10^{-9}$	$-2.21\cdot10^{3}$
Gas, natural, feedstock, 35 MJ per m³, in ground	Raw	$0.01\cdot10^{-9}$	$-2.52\cdot10^{3}$	$0.01\cdot10^{-9}$	$-2.93\cdot10^{2}$	$0.01\cdot10^{-9}$	$-2.58\cdot10^{3}$	$0.01\cdot10^{-9}$	$-2.24\cdot10^{3}$
Gas, natural, in ground	Raw	x	x	x	x	$1.69\cdot10^{2}$	x	$1.40\cdot10^{3}$	x
Oil, crude, 42.6 MJ per kg, in ground	Raw	$6.53\cdot10^{2}$	$-4.91\cdot10^{2}$	$1.42\cdot10^{3}$	$-7.23\cdot10^{1}$	$1.32\cdot10^{3}$	$-5.59\cdot10^{2}$	$0.01\cdot10^{-9}$	$-9.25\cdot10^{2}$
Oil, crude, 42.7 MJ per kg, in ground	Raw	$2.22\cdot10^{4}$	x	$5.12\cdot10^{2}$	x	$1.82\cdot10^{4}$	x	$1.68\cdot10^{4}$	x
Oil, crude, feedstock, 41 MJ per kg, in ground	Raw	$0.01\cdot10^{-9}$	$-2.52\cdot10^{3}$	$0.01\cdot10^{-9}$	$-2.76\cdot10^{2}$	$0.01\cdot10^{-9}$	$-2.56\cdot10^{3}$	$0.01\cdot10^{-9}$	$-3.36\cdot10^{3}$
Oil, crude, in ground	Raw	x	x	x	x	$4.13\cdot10^{1}$	x	$8.04\cdot10^{2}$	x
Remaining substances	x	$8.60\cdot10^{0}$	$0.00\cdot10^{0}$	$0.00\cdot10^{0}$	$0.00\cdot10^{0}$	$1.24\cdot10^{1}$	$0.00\cdot10^{0}$	$7.11\cdot10^{0}$	$0.00\cdot10^{0}$
**TOTAL**		**$2.55\cdot10^{4}$**	**$-7.53\cdot10^{3}$**	**$2.63\cdot10^{3}$**	**$-8.61\cdot10^{2}$**	**$2.24\cdot10^{4}$**	**$-7.71\cdot10^{3}$**	**$2.12\cdot10^{4}$**	**$-8.26\cdot10^{3}$**
